# A novel Pulp Caries GAN multi loss GAN with new pulp inspired metaheuristics for pediatric dental caries detection and segmentation

**DOI:** 10.1038/s41598-025-28459-8

**Published:** 2026-01-08

**Authors:** Amira Abdelhafeez Elkhatib, Mostafa Elbaz, Riham Sobhy Soliman, Mona Elshirbini Hafez

**Affiliations:** 1 Pediatric Dentistry and Dental Public Health, Faculty of Oral and Dental Medicine, Kafrelsheikh University, Kafrelsheikh, Egypt; 2 Department of Computer Science, Faculty of Computers and Informatics, Kafrelsheikh University, Kafrelsheikh, Egypt; 3 Pediatric Dentistry and Dental Public Health, Faculty of Dentistry, Alexandria University, Alexandria, Egypt; 4 Faculty of Oral and Dental Medicine, Kafrelsheikh University, Kafrelsheikh, Egypt

**Keywords:** Dental caries, Pediatric imaging, Generative adversarial networks, Biomimetic optimization, Medical image synthesis, Computational biology and bioinformatics, Data processing, Image processing

## Abstract

Early detection of dental caries in pediatric populations remains challenging due to limited annotated datasets and the subtle manifestation of incipient lesions. This study introduces Pulp-Caries-GAN, a novel generative adversarial network incorporating a biomimetic optimization strategy for high-fidelity synthetic dental image generation. The framework integrates a multi-loss architecture combining adversarial, pixel-wise, perceptual, and structural similarity losses with a pulp-inspired metaheuristic function that models neurophysiological dynamics of dental pulp tissue to preserve anatomical coherence. The optimization strategy employs spatially-adaptive regularization through an anatomical masking mechanism that enforces tissue-specific constraints based on diagnostic importance. Experimental validation was conducted on a pediatric dental panoramic dataset comprising 193 annotated images from 106 patients aged 2–13 years. The results demonstrate superior image synthesis quality compared to conventional GAN architectures, achieving a Fréchet Inception Distance of 154.87, Inception Score of 80.12, and Peak Signal-to-Noise Ratio of 80.04. Integration of synthetic images generated by Pulp-Caries-GAN significantly enhanced segmentation performance across multiple U-Net variants. The Hierarchical Dense U-Net achieved optimal results with a Dice coefficient of 95.12%, accuracy of 95.65%, precision of 95.32%, and recall of 93.7%. Ablation studies confirmed the critical role of the pulp-inspired loss component and anatomical masking in maintaining structural integrity while reducing artifacts in synthetic images. Clinical validation by five board-certified pediatric dentists revealed that 87% of synthetic images were clinically indistinguishable from real radiographs, with 94% of synthetic lesions exhibiting anatomically correct progression patterns. These findings demonstrate the efficacy of biomimetic optimization approaches in medical image synthesis and establish a robust framework for automated pediatric dental caries detection with potential for clinical translation.

**Supplementary Information:**

The online version contains supplementary material available at 10.1038/s41598-025-28459-8.

## Introduction

Pediatric dental caries represents a multifactorial pathological process affecting approximately 60–90% of children globally, constituting the most prevalent chronic disease in this demographic and posing significant challenges to public health systems worldwide^1–3^. The pathogenesis of dental caries in pediatric populations involves complex interactions between cariogenic bacteria, dietary carbohydrates, host factors, and temporal dynamics, with early childhood caries demonstrating particularly aggressive progression patterns that can lead to extensive tooth destruction within months of eruption^4,5^. The critical importance of early detection lies in the exponential relationship between intervention timing and treatment complexity, where detection at the initial demineralization stage enables non-invasive remineralization therapies, while delayed diagnosis necessitates invasive restorative procedures with associated psychological trauma and increased healthcare costs.

Contemporary diagnostic methodologies in pediatric dentistry rely predominantly on visual-tactile examination supplemented by bitewing radiography, approaches that demonstrate inherent limitations in sensitivity and specificity for incipient lesion detection^6–10^. Visual examination techniques, while non-invasive and cost-effective, exhibit substantial inter-examiner variability and limited capability for detecting subsurface demineralization, particularly in approximal surfaces where 40–60% of pediatric caries initiate. Radiographic imaging, though providing enhanced visualization of interproximal lesions, involves ionizing radiation exposure concerns in developing patients and demonstrates reduced sensitivity for early enamel caries due to the substantial mineral loss threshold required for radiographic visibility^11,12^. These diagnostic limitations contribute to significant epidemiological underestimation of caries prevalence and delayed therapeutic intervention, emphasizing the critical need for enhanced detection methodologies.

The emergence of artificial intelligence, particularly deep learning architectures, has demonstrated transformative potential in medical imaging applications, with convolutional neural networks achieving superior performance in pathological pattern recognition tasks across multiple medical domains^13,14^. U-Net architectures have shown particular efficacy in biomedical image segmentation applications, demonstrating the capability to learn hierarchical feature representations that capture subtle morphological variations indicative of pathological processes. However, the clinical translation of deep learning methodologies in pediatric dental caries detection faces substantial challenges related to dataset limitations, where the scarcity of high-quality annotated images constrains model generalization and clinical applicability^15^. Pediatric dental imaging datasets are inherently limited due to ethical considerations regarding radiation exposure, patient cooperation challenges, and the specialized expertise required for accurate caries annotation, creating a fundamental bottleneck in algorithm development.

Generative Adversarial Networks present a paradigm-shifting approach to address dataset limitations through synthetic image generation, offering the potential to augment training datasets while preserving the statistical properties and clinical relevance of original data^16,17^. However, conventional GAN architectures face significant challenges in medical imaging applications, including mode collapse, training instability, and the generation of clinically irrelevant artifacts that can compromise downstream diagnostic performance^18,19^. The preservation of anatomical accuracy and pathological fidelity in synthetic dental images requires specialized optimization strategies that transcend traditional adversarial training approaches, necessitating the integration of domain-specific knowledge and biologically-informed regularization mechanisms^20^.

This investigation introduces Pulp-Caries-GAN, a novel generative adversarial network architecture incorporating a biomimetic metaheuristic optimization strategy inspired by the neurophysiological dynamics of dental pulp tissue. The proposed framework addresses the fundamental challenges of synthetic medical image generation through a multi-loss function architecture that integrates adversarial training with pixel-wise reconstruction, perceptual similarity, and structural coherence constraints. The pulp-inspired optimization strategy employs spatially-adaptive regularization mechanisms that enforce anatomically-informed constraints based on tissue-specific diagnostic importance, thereby preserving the clinical relevance of synthetic images while enhancing dataset diversity for deep learning model training.

The contributions of this paper as the follow:

1. A novel Pulp-Caries-GAN architecture for pediatric dental caries synthesis that addresses dataset limitations through high-fidelity image generation. The framework achieves superior performance with FID score of 154.87 and IS score of 80.12 compared to existing GAN architectures.
2. A pulp-inspired metaheuristic optimization strategy that models neurophysiological dynamics of dental pulp tissue to enhance synthetic image quality. This biomimetic approach preserves anatomical accuracy and structural coherence in generated pediatric dental images.
3. A multi-loss function framework integrating adversarial, pixel-wise, perceptual, and PSNR losses with pixel-coherence preservation mechanisms. The approach effectively reduces artifacts while maintaining spatial relationships and structural continuity in synthetic images.
4. Empirical validation demonstrating enhanced segmentation performance across multiple U-Net architectures when augmented with Pulp-Caries-GAN. The best performing model achieves 95.12% Dice Score, 95.65% accuracy, 95.32% precision, and 93.7% recall.

### Advantages of Pulp-Caries-GAN Over Other Inspired Metaheuristics as a Loss Function.

- Introduces spatial neighborhood constraints to reduce artifacts, distortions, and pixel inconsistencies, resulting in higher-quality synthetic images for pediatric dental caries detection.
- Reduces overfitting and mode collapse, ensuring that GAN-generated images are more clinically accurate and applicable across diverse pediatric datasets.
- Regulates gradient flow, preventing unstable training behavior and accelerating convergence compared to traditional GAN loss functions.
- Reduces the need for large annotated datasets, making AI-powered dental diagnostics more accessible in clinics with limited data.
- Unlike general bio-inspired metaheuristics (e.g., genetic algorithms, swarm intelligence), the pulp-inspired loss function is specifically designed for dental image augmentation, ensuring greater clinical relevance.

The organization of the paper are as the follow: Sect. 1 introduces the clinical significance and challenges of pediatric dental caries detection. Section 2 reviews related work in dental imaging, deep learning architectures, and GAN applications. Section 3 details the Pulp-Caries-GAN methodology, multi-loss framework, and pulp-inspired optimization strategy. Section 4 presents experimental results and performance evaluations. Section 5 discusses clinical implications and expert validation. Section 6 concludes with key findings and future research directions.

## The related work

This part of paper introduces the different architectures can be used on the segmentation of the children dental caries, the different augmentation techniques using GANs for dental images augmentations, research gap and research questions.

###  U-Net architectures in dental caries detection

Recent advancements in U-Net^21^ architectures have significantly improved the detection of dental caries in pediatrics such as U-Net++, which incorporates nested skip pathways to enhance feature propagation, and U-Net3+^22^, which utilizes attention mechanisms for better segmentation of complex structures. The Attention U-Net^23^ focuses on relevant features while suppressing noise, facilitating the identification of subtle lesions. Similarly, ResU-Net combines residual connections to promote better gradient flow, while 3D U-Net^24^ extends capabilities to volumetric data, making it suitable for CT and MRI scans. Nested U-Net features a nested skip pathway architecture for improved accuracy across different resolutions. The Dilated U-Net employs dilated convolutions to capture larger structures, and Multi-Res U-Net^25^ processes features at multiple resolutions, enhancing detection of various degrees of caries. The Spatial Attention U-Net^26^ emphasizes informative regions, improving segmentation accuracy, while the Variational U-Net combines U-Net with variational autoencoder principles for probabilistic outputs. Additionally, Residual Attention U-Net integrates residual connections with attention mechanisms, allowing the model to focus on important features while maintaining gradient flow. These architectures represent a significant evolution in deep learning techniques aimed at enhancing pediatric dental health outcomes.

Eun Young Park et al.^27^ conducted a significant study that evaluated deep learning algorithms for caries detection through the segmentation of tooth surfaces using intraoral photographic images. In their prospective research, they collected 2,348 images from 445 participants at a university medical center over a period from October 2020 to December 2021. The dataset was divided into training (1,638), validation (410), and test (300) subsets to rigorously assess the performance of the models. Employing convolutional neural networks (CNNs), including U-Net, ResNet-18, and Faster R-CNN, they aimed to classify and localize carious lesions effectively. Their results indicated a significant enhancement in classification accuracy, achieving an accuracy of 0.813 and an area under the receiver operating characteristic curve (AUC) of 0.837, compared to earlier values of 0.758 and 0.731. Additionally, the localization of carious lesions showed improved sensitivity and average precision, with values increasing from 0.890 to 0.865 and 0.889 to 0.868, respectively.

Shunv Ying et al.^28^ developed an advanced deep learning network that leverages contemporary methodologies in biomedical imaging. The primary objective of their study was to enhance the segmentation of dental caries in clinically obtained X-ray images. Their proposed network builds upon the established architecture of U-Net by incorporating skip connections while creatively integrating vision transformers, dilated convolutions, and feature pyramid fusion techniques. These enhancements significantly improved the model’s ability to extract multi-scale and global features. The network was trained on a self-collected and augmented dataset of tooth X-ray images, and its performance was rigorously evaluated using metrics such as dice similarity and pixel classification precision. The results demonstrated an impressive average dice similarity coefficient of 0.7487 and an average pixel classification precision of 0.7443 on the test dataset.

A significant study by Shuaa S. Alharbi et al.^29^ explored the application of various U-Net models to dental panoramic X-ray images for the detection of caries lesions. They utilized the Detection, Numbering, and Segmentation Panoramic Images (DNS) dataset, comprising 1,500 panoramic X-ray images sourced from Ivisionlab. The primary objective of their work was to enhance the DNS dataset by accurately identifying cavities within the panoramic images and generating binary ground truth annotations for model evaluation. These annotations were meticulously reviewed by experts to ensure their accuracy and reliability.

The researchers expanded the DNS dataset by detecting cavities and creating corresponding binary ground truths. They then implemented U-Net, U-Net++, and U-Net3 + architectures on the expanded dataset to learn hierarchical features and improve the delineation of cavity boundaries. Their findings revealed that U-Net3 + achieved superior performance, attaining a testing accuracy of 95%, thereby demonstrating the effectiveness of deep learning models in enhancing caries detection within dental panoramic radiographs. This study highlights the critical role of comprehensive datasets and advanced segmentation techniques in improving diagnostic accuracy and clinical outcomes in dentistry.

Jian Wu^30^ introduced a novel deep learning architecture named Caries-Net. This architecture aims to delineate various degrees of caries from panoramic radiographs. The researchers compiled a high-quality dataset consisting of 3,127 precisely annotated caries lesions, categorized into shallow, moderate, and deep caries. Caries-Net is constructed as a U-shaped network enhanced with a full-scale axial attention module, specifically designed to segment these three types of caries from oral panoramic images.

The performance of Caries-Net was rigorously evaluated against several baseline methods, demonstrating superior segmentation capabilities. The experimental results indicated that Caries-Net achieved a mean Dice coefficient of 93.64% and an accuracy of 93.61% in distinguishing the different levels of caries. This study highlights the potential of advanced deep learning techniques to improve the accuracy and efficiency of caries diagnosis, aligning with ongoing efforts to enhance diagnostic methodologies in pediatric dentistry and beyond. Table 1 shows summarize the uses of deep learning architecture in dental caries detection advantages, disadvantages and research gaps.

**Table 1:** Recent work in dental caries detection using deep learning architecture.

Study	Methodology	Materials Used	Advantages	Disadvantages	Research Gaps
Eun Young Park et al.^27^	Deep learning algorithms (CNNs: U-Net, ResNet-18, Faster R-CNN) for segmentation of tooth surfaces	2,348 intraoral photographs from 445 participants	High accuracy (0.813) and AUC (0.837) for caries detection	Relies on manual image collection; potential for bias in selection	Enhances the dataset with more comprehensive annotations and ground truths
Shunv Ying et al.^28^	Advanced U-Net architecture with skip connections, vision transformers, dilated convolutions	Self-collected and augmented dataset of tooth X-ray images	Improved feature extraction for caries segmentation (Dice = 0.7487, Precision = 0.7443)	Limited to X-ray images; may struggle with varying image quality	Focuses on panoramic radiographs, expanding the application of deep learning in different imaging modalities
Shuaa S. Alharbi et al.^29^	Implementation of U-Net, U-Net++, and U-Net3 + for cavity detection	DNS dataset with 1,500 panoramic X-ray images	High testing accuracy (95%) with enhanced boundary delineation	Potential overfitting due to limited dataset size; reliance on expert review	Expands the dataset and improves the accuracy of binary ground truth annotations
Jian Wu^30^	U-shaped network (CariesNet) with axial attention module for caries segmentation	High-quality dataset of 3,127 annotated caries lesions	High mean Dice coefficient (93.64%) and accuracy (93.61%)	Complexity of the model may limit generalizability; requires extensive training data	Introduces a novel architecture that combines segmentation techniques with attention mechanisms for improved accuracy

### GANs architectures for dental caries augmentation

Various architectures of Generative Adversarial Networks (GANs) have emerged as effective tools for augmenting dental caries images in children, significantly addressing the challenges posed by limited datasets. Standard GAN serves as the foundational model, capable of generating synthetic images that enhance existing data. Deep Convolutional GAN (DCGAN)^31^ improves the realism of generated images through the use of convolutional layers, making it particularly suitable for dental applications. Pix2Pix GAN excels in image-to-image translation tasks, enabling the synthesis of dental caries images based on input images with varying quality and resolution. CycleGAN is advantageous for transforming images between different domains without the need for paired datasets, which is particularly useful for augmenting diverse dental imaging modalities. StyleGAN offers advanced control over image styles and features, facilitating the generation of high-fidelity dental images that can depict various stages and conditions of caries. Conditional GAN (cGAN) allows for targeted image augmentation by conditioning the generation process on specific labels, such as the severity of caries, resulting in tailored synthetic images for training deep learning models. Wasserstein GAN (WGAN)^32^ enhances training stability and image quality by employing a Wasserstein distance metric, which can lead to more realistic dental image generation. MCI-GAN (a novel GAN with identity blocks inspired by menstrual cycle behavior for missing pixel imputation)^20^ incorporates multiple conditions to improve the diversity and specificity of generated images. Additionally, 8-Connected GAN^33^ utilizes an 8-connected topology to enhance spatial coherence in generated images, crucial for the detailed structures found in dental imaging. Identity GAN focuses on preserving the identity of original inputs during image generation, ensuring that critical diagnostic features remain intact. Lastly, Gsip GAN (GAN-based sperm-inspired pixel imputation)^34^addresses spatial invariance in image generation, maintaining consistent features across different spatial configurations.

### The research gaps in

Despite significant advancements in the use of deep learning techniques for dental caries detection, several critical research gaps remain unaddressed. First, many existing studies rely on limited datasets, which may not fully capture the variability in caries presentations across diverse populations and imaging modalities. This can lead to overfitting and reduced generalizability of the models. Additionally, while some methodologies have shown promise in specific imaging contexts, there is a lack of comprehensive approaches that effectively integrate various imaging techniques, such as panoramic radiography and intraoral photography. Furthermore, the reliance on expert-reviewed annotations, while valuable, can introduce biases and may not always reflect real-world clinical scenarios. Our research aims to fill these gaps by expanding existing datasets with robust annotations, exploring the application of advanced segmentation techniques across different imaging modalities, and developing models that are more adaptable to the complexities of clinical practice. This holistic approach seeks to enhance the accuracy and efficiency of caries diagnosis, ultimately improving patient outcomes in pediatric dentistry. The research gaps summarized in this points.

1. Existing studies often rely on small and homogenous datasets, which may not capture the full variability in caries presentations across diverse populations.
2. The limited scope of many models can lead to overfitting, reducing their generalizability to real-world clinical scenarios.
3. Many methodologies focus on specific imaging techniques, lacking comprehensive approaches that integrate various modalities, such as panoramic radiography and intraoral photography.
4. Reliance on expert-reviewed annotations can introduce biases, which may not accurately reflect everyday clinical conditions.
5. There is a lack of comprehensive, high-quality annotations that enhance the training and evaluation of deep learning models.
6. Current models often do not account for the complexities encountered in clinical practices, limiting their practical application.
7. There is a need for improved segmentation techniques that combine various deep learning methodologies to enhance diagnostic accuracy.

### The research questions

1. How can we enhance the quality and diversity of datasets for dental caries detection to improve model generalizability?
2. What advanced deep learning architectures can be developed to integrate multiple imaging modalities effectively for caries diagnosis?
3. In what ways can we improve annotation processes to reduce bias and ensure that the training data accurately reflects real-world clinical scenarios?
4. How do different deep learning models compare in their ability to segment various degrees of caries from panoramic radiographs?
5. What are the optimal techniques for combining segmentation and attention mechanisms to improve the accuracy of caries detection?
6. How can we ensure that the developed models are robust enough to handle the complexities and variability encountered in clinical practice?
7. What metrics can be utilized to comprehensively evaluate the performance of deep learning models in the context of caries detection?

## Material and method

### Experimental dataset and preprocessing

**Fig. 1:**
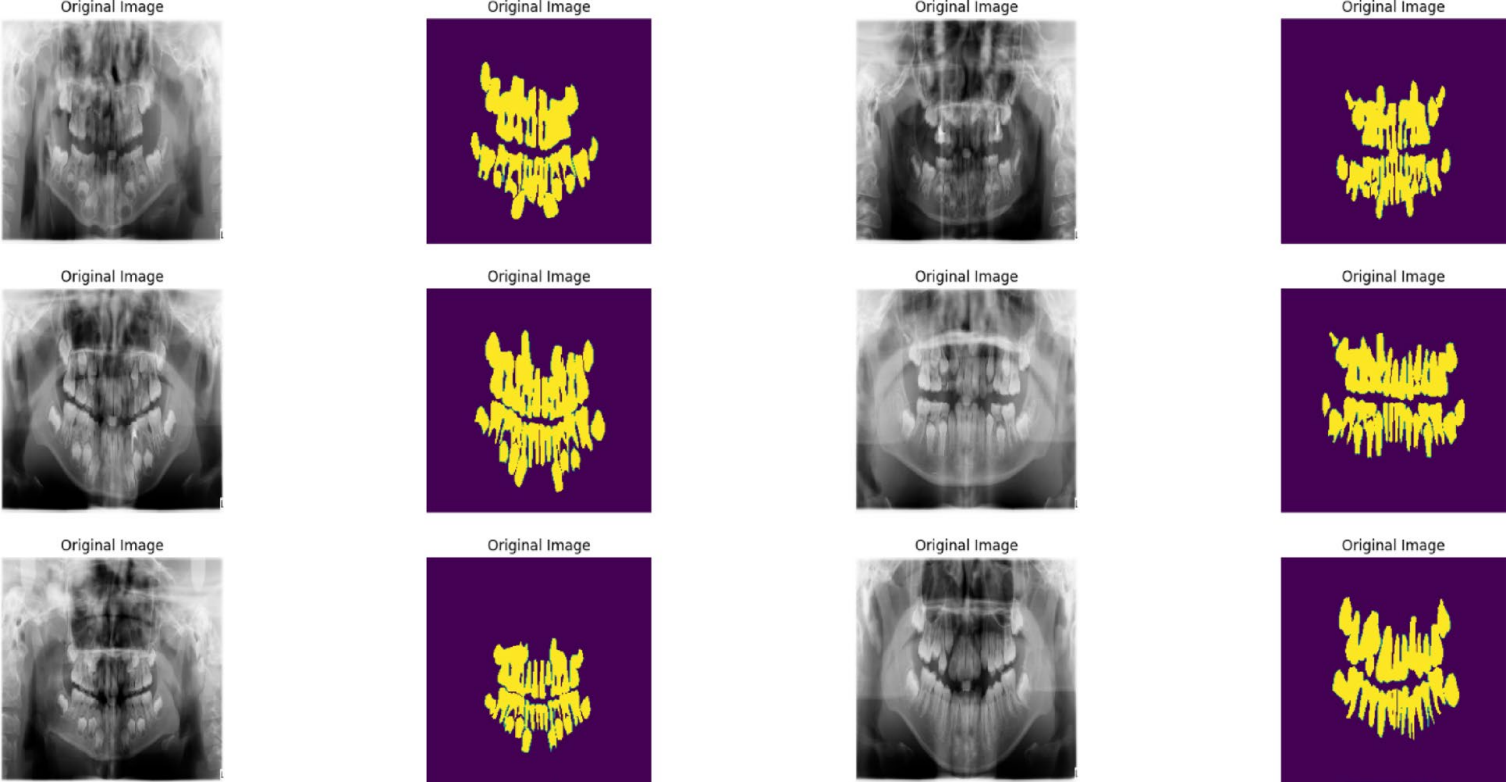
samples of images from dental panoramic dataset.

This study utilizes a pediatric dental panoramic radiograph dataset comprising 193 annotated images from 106 patients aged 2–13 years, collected at Hangzhou Xiasha Dental Hospital between March and June 2022. The dataset includes 123 panoramic radiographs and 93 supplementary dental images, with annotations created using EISeg and LabelMe platforms for caries segmentation and dental disease detection. Each image underwent preprocessing including normalization to [0,1] range, histogram equalization for contrast enhancement, and patch extraction (256 × 256) with 50% overlap for training purposes. The limited dataset size (193 images) necessitates robust data augmentation strategies to enhance model generalizability and address the fundamental challenge of annotated data scarcity in pediatric dental imaging applications. Figure 1 shows examples of used images from the dataset.

### The Pulp-Caries-GAN framework

The Pulp-Caries-GAN framework represents a groundbreaking advancement in pediatric dental imaging by employing a novel Generative Adversarial Network (GAN) for the augmentation of dental panoramic datasets. This innovative architecture integrates a unique multi-loss function framework, combining adversarial loss, pixel-wise loss, perceptual loss, and structural similarity index (SSIM) loss, to generate high-fidelity synthetic dental images. Central to its methodology is a pulp-inspired metaheuristic optimization strategy that mimics the neurophysiological dynamics of dental pulp tissue. This approach not only enhances the spatial coherence and structural continuity of the generated images but also effectively addresses the critical issue of limited annotated datasets in pediatric dentistry. By augmenting existing datasets with realistic synthetic images, Pulp-Caries-GAN significantly improves the training of deep learning models, leading to more accurate and reliable detection of dental caries in children. This advancement holds the potential to facilitate earlier interventions and improve overall pediatric dental health outcomes.

**Fig. 2:**
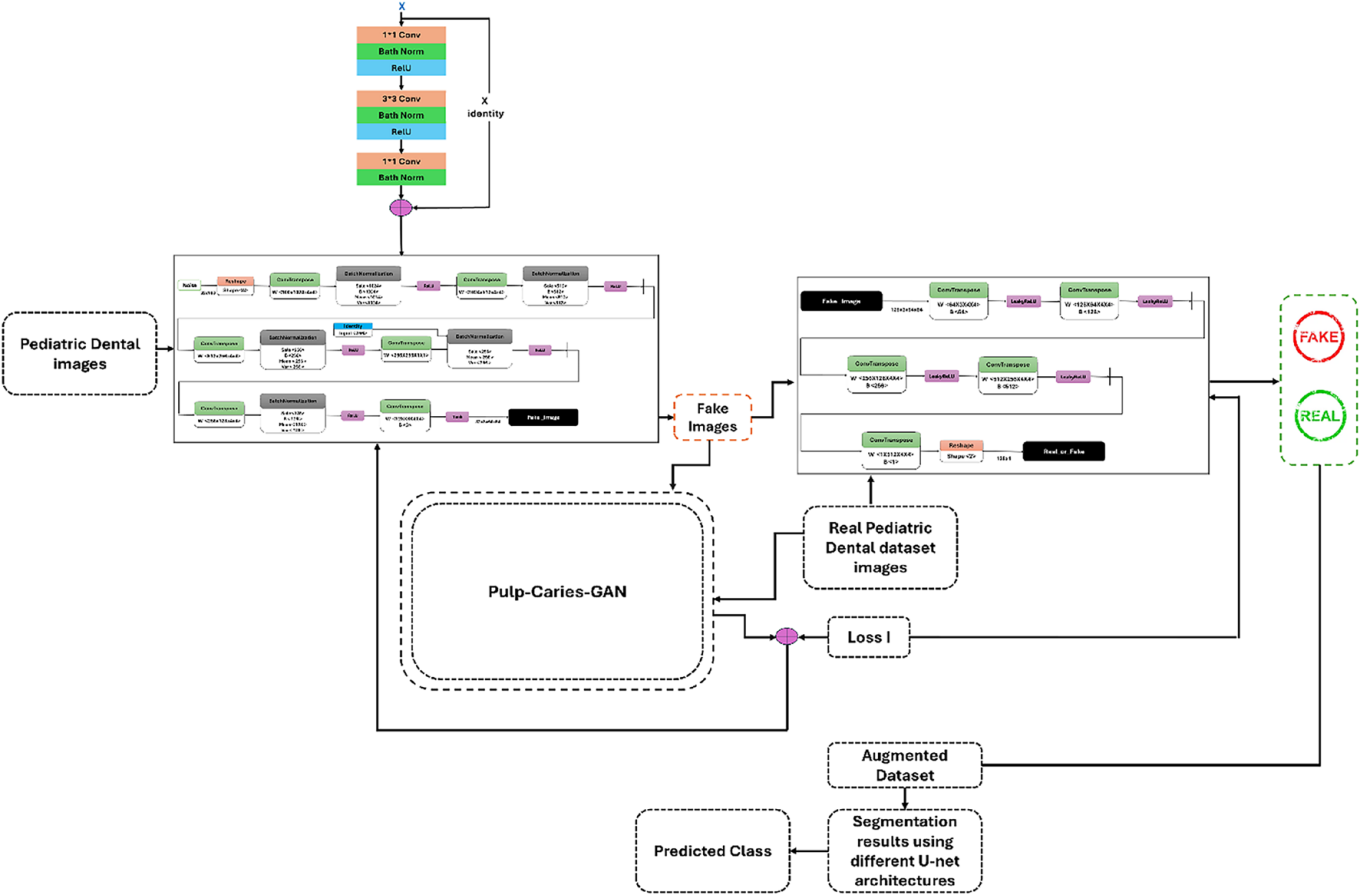
Block diagram of the methodology.

The architecture of the Novel Pulp-Caries-GAN is designed to enhance the accuracy of pediatric dental caries detection through advanced image synthesis. At its core, this framework consists of two primary components: a Generator (G) and a Discriminator (D), which operate in a competitive manner to improve the quality of synthetic dental images. The Generator utilizes a multi-loss function approach that integrates adversarial loss, pixel-wise loss, perceptual loss, and SSIM loss, ensuring high fidelity and realism in the generated images. Additionally, the architecture incorporates a pulp-inspired optimization strategy that simulates the neurophysiological dynamics of dental pulp tissue, enabling the generation of images with preserved structural characteristics. A novel aspect of this architecture is the evaluation of generated images against specific criteria defined by the pulp loss function; only those images that meet these criteria are included in the augmented dataset, thereby enhancing the overall quality of training data for deep learning models. This systematic approach is outlined in Algorithm 1, which details the training process and the integration of the multi-loss function within the architecture. Figure 2 shows the block diagram of the methodology.

**Algorithm 1:**
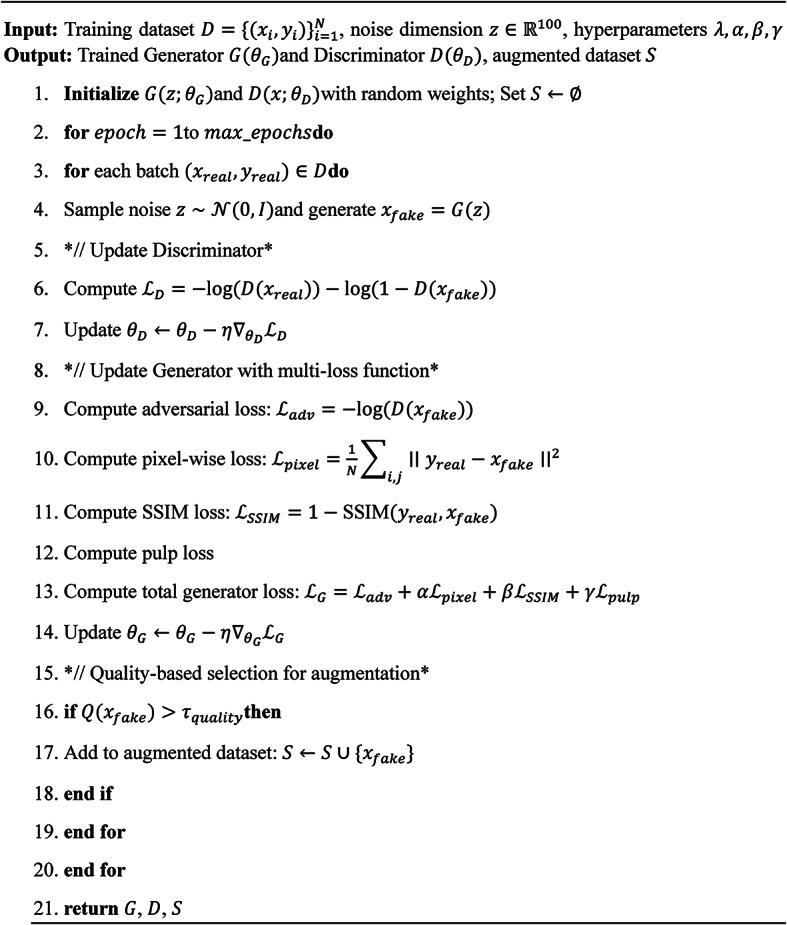
Training procedure for Pulp-Caries-GAN.

The generator network follows a deep convolutional architecture designed to transform random noise vectors into realistic dental caries images. As shown in Table 2, the network begins with a 100-dimensional noise vector that undergoes a dense transformation and reshaping operation, resulting in a 4 × 4 × 1024 feature map. The architecture then employs a series of upsampling operations through transposed convolutions, progressively increasing the spatial dimensions while reducing the feature depth. A notable aspect of this generator is the incorporation of Dense Blocks at multiple stages (layers 6, 9, 12, and 15), which enhance feature reuse and improve gradient flow throughout the network. Each upsampling stage is followed by batch normalization and ReLU activation, promoting stable training and non-linear representation capabilities. The final layers (18–19) refine the generated features into a 128 × 128 × 3 RGB image using standard convolutions, with a tanh activation function normalizing the output values between − 1 and 1.

**Table 2:** Generator network architecture.

Layer	Type	Parameters	Output Shape	Activation	Notes
1	Input	-	(100, 1, 1)	-	Random noise vector z
2	Dense	6,553,600	(100, 4, 4, 1024)	-	Fully connected & reshaped
3	BatchNorm	-	(4, 4, 1024)	ReLU	Normalization
4	Conv2DTranspose	26,214,400	(8, 8, 512)	-	Upsampling, stride = 2
5	BatchNorm	-	(8, 8, 512)	ReLU	Normalization
6	Dense Block 1	4,718,592	(8, 8, 512)	ReLU	3 × 3 conv, growth rate = 64
7	Conv2DTranspose	6,553,600	(16, 16, 256)	-	Upsampling, stride = 2
8	BatchNorm	-	(16, 16, 256)	ReLU	Normalization
9	Dense Block 2	2,359,296	(16, 16, 256)	ReLU	3 × 3 conv, growth rate = 32
10	Conv2DTranspose	1,638,400	(32, 32, 128)	-	Upsampling, stride = 2
11	BatchNorm	-	(32, 32, 128)	ReLU	Normalization
12	Dense Block 3	1,179,648	(32, 32, 128)	ReLU	3 × 3 conv, growth rate = 16
13	Conv2DTranspose	409,600	(64, 64, 64)	-	Upsampling, stride = 2
14	BatchNorm	-	(64, 64, 64)	ReLU	Normalization
15	Dense Block 4	589,824	(64, 64, 64)	ReLU	3 × 3 conv, growth rate = 8
16	Conv2DTranspose	49,152	(128, 128, 32)	-	Upsampling, stride = 2
17	BatchNorm	-	(128, 128, 32)	ReLU	Normalization
18	Conv2D	9,216	(128, 128, 16)	ReLU	Refinement
19	Conv2D	432	(128, 128, 3)	Tanh	Output RGB image

The discriminator network, detailed in Table 3, employs a conventional classification architecture to distinguish between real and generated dental caries images. Starting with a 128 × 128 × 3 input image, the network progressively reduces spatial dimensions while increasing feature depth through a series of convolutional layers with stride = 2. The architecture follows a pattern of strided convolutions for downsampling, followed by same-padding convolutions for feature extraction, with batch normalization applied after each feature extraction step. LeakyReLU activations with a negative slope of 0.2 are used throughout to prevent the dying ReLU problem and maintain gradient flow. After several convolutional blocks that reduce the input to an 8 × 8 × 512 representation, the features are flattened and processed through fully connected layers with dropout regularization (rate = 0.4) to prevent overfitting. The network culminates in a single-output sigmoid activation that produces a probability score indicating whether the input image is real or generated.

**Table 3:** Discriminator network architecture.

Layer	Type	Parameters	Output Shape	Activation	Notes
1	Input	-	(128, 128, 3)	-	Dental caries image
2	Conv2D (k3n64)	1,728	(64, 64, 64)	LeakyReLU(0.2)	Stride = 2, padding = same
3	Conv2D (k3n64)	36,864	(64, 64, 64)	LeakyReLU(0.2)	Stride = 1, padding = same
4	BatchNorm	-	(64, 64, 64)	-	Normalization
5	Conv2D (k3n128)	73,728	(32, 32, 128)	LeakyReLU(0.2)	Stride = 2, padding = same
6	Conv2D (k3n128)	147,456	(32, 32, 128)	LeakyReLU(0.2)	Stride = 1, padding = same
7	BatchNorm	-	(32, 32, 128)	-	Normalization
8	Conv2D (k3n256)	294,912	(16, 16, 256)	LeakyReLU(0.2)	Stride = 2, padding = same
9	Conv2D (k3n256)	589,824	(16, 16, 256)	LeakyReLU(0.2)	Stride = 1, padding = same
10	BatchNorm	-	(16, 16, 256)	-	Normalization
11	Conv2D (k3n512)	1,179,648	(8, 8, 512)	LeakyReLU(0.2)	Stride = 2, padding = same
12	Conv2D (k3n512)	2,359,296	(8, 8, 512)	LeakyReLU(0.2)	Stride = 1, padding = same
13	BatchNorm	-	(8, 8, 512)	-	Normalization
14	Flatten	-	(32,768)	-	Flatten to 1D
15	Dense	16,384	(512)	LeakyReLU(0.2)	Fully connected
16	Dropout	-	(512)	-	Dropout rate = 0.4
17	Dense	513	(1)	Sigmoid	Binary classification

The training procedure for the dental caries GAN employed a sophisticated configuration optimized for medical imaging applications. As detailed in Table 4, the network utilized a specialized loss function combining traditional Binary Cross-Entropy with a novel Pulp Loss component specifically designed to enhance dental feature fidelity. The Adam optimizer was selected with carefully tuned hyperparameters (learning rate of 0.0002 and β1 of 0.5) to ensure stable convergence while avoiding mode collapse. Training was conducted with asymmetric batch sizes—64 for dataset A and 32 for dataset B—accommodating the different characteristics of real and fake image distributions. The model underwent a comprehensive 200-epoch training regimen with a strategic learning rate decay implemented after the 100th epoch to fine-tune the generator’s output quality. All dental images were processed at a consistent 128 × 128 RGB resolution to balance computational efficiency with diagnostic detail preservation. Domain-specific data augmentation techniques including rotation, flipping, and brightness adjustments were applied to expand the training dataset while maintaining clinically relevant variations. Additionally, identity mapping was incorporated into the architecture to preserve critical dental structural details, ensuring the generated images maintained anatomical accuracy required for diagnostic applications.

**Table 4:** Training configuration.

Parameter	Value	Notes
Loss function	Binary Cross-Entropy + Novel Pulp Loss	Combined loss for dental specificity
Optimizer	Adam	Learning rate: 0.0002, β1: 0.5
Batch size	A-64, B-32	For real/fake images respectively
Training epochs	200	With learning rate decay after 100 epochs
Image resolution	128 × 128	RGB dental images
Data augmentation	Rotation, flip, brightness	Specific to dental imaging
Identity mapping	Applied	To preserve dental structure details

### Novel pulp used as a metaheuristic loss function

The proposed pulp-inspired loss function draws theoretical inspiration from the spatial organization and response mechanisms of dental pulp tissue, while maintaining mathematical rigor through established optimization principles. Dental pulp exhibits spatially correlated cellular responses to stimuli, where cellular activities are influenced by neighboring cell states through gap junctions and intercellular communication pathways. This biological observation motivates a spatial regularization approach that enforces neighborhood coherence in synthetic image generation.

The mathematical formulation translates this biological principle into a differentiable optimization framework that preserves local spatial relationships while allowing global image synthesis. Unlike traditional spatial regularization methods such as total variation (TV) regularization or bilateral filtering, the pulp-inspired approach incorporates anatomically-informed weighting that adapts to dental tissue characteristics.

The Novel Pulp Loss function $\:{P}_{loss}$ is formally defined as a weighted spatial coherence term that minimizes local pixel variations while preserving diagnostically relevant features as Eq. (1).

1 $$P_{{loss}} = ~{}_{1}^{n} Y\sum \left\{ {\left( {i,j} \right) \in \Omega } \right\}\sum \left\{ {p \in N\left( {i,j} \right)} \right\}w\left( {d_{{\left\{ {i,j,p} \right\}}} } \right)\cdot~\left| {\left| {G\left( z \right)_{{\left\{ {i,j} \right\}}} - ~G\left( z \right)_{p} } \right|} \right|^{2} \cdot~M\left( {i,j,p} \right)$$

where Ω represents the image domain, $\:N\left(i,j\right)$ denotes the 8-connected neighborhood of $\:pixel\:\left(i,j\right),\:G{\left(z\right)}_{\left\{i,j\right\}}$ is the generated pixel intensity at coordinates (i, j), and λ is the regularization parameter.

The distance weighting function $\:w\left({d}_{\left\{i,j,p\right\}}\right)$follows a Gaussian kernel formulation as Eq. (2), where $\:{d}_{\left\{i,j,p\right\}}$ represents the Euclidean distance between pixels (i, j) and p, and σ controls the spatial influence radius. This formulation ensures that closer pixels have stronger influence, mimicking the decreasing intercellular communication strength with distance observed in biological tissues.

2 $$\:w\left({d}_{\left\{i,j,p\right\}}\right)=\text{e}\text{x}\text{p}\left(-\frac{{d}_{\left\{i,j,p\right\}}^{2}}{2{\sigma\:}^{2}}\right)$$

The anatomical masking function $\:M\left(i,j,p\right)$ serves as a spatially-adaptive weight that emphasizes diagnostically relevant regions based on dental anatomy priors as Eq. (3). where $\:{M}_{enamel},\:{M}_{dentin},\:and\:{M}_{boundary}$ represent binary masks for enamel regions, dentin regions, and tissue boundaries respectively, with α, β, γ as learned or predefined weighting coefficients.

3 $$M\left( {i,j,p} \right) = \alpha \cdot M_{{enamel\left( {i,j,p} \right)}} + \beta \cdot M_{{dentin\left( {i,j,p} \right)}} + \gamma \cdot M_{{boundary\left( {i,j,p} \right)}}$$

During training, $\:M\left(i,j,p\right)$ is computed dynamically using a pre-trained dental anatomy segmentation network that identifies tissue types in real images. For synthetic images, the mask is propagated from the corresponding real image guidance. This mechanism ensures that the loss function adapts its emphasis based on the clinical significance of different anatomical regions.

### Biological justification and empirical validation of Pulp-Inspired loss

#### Neurophysiological foundation

The pulp-inspired loss function derives its theoretical foundation from well-documented neurophysiological mechanisms observed in dental pulp tissue. Dental pulp represents a highly specialized connective tissue containing a complex network of odontoblastic cells, nerve fibers, and vascular elements that exhibit coordinated cellular responses to external stimuli. The biological rationale for our mathematical formulation stems from three key physiological observations documented in dental neuroscience literature.

First, odontoblastic cells in dental pulp demonstrate spatially-correlated activity patterns through gap junction-mediated intercellular communication. These connexin-43 gap junctions enable direct cytoplasmic continuity between adjacent cells, facilitating the propagation of calcium signals and small molecules across cellular networks. Experimental studies using calcium imaging techniques have demonstrated that mechanical or thermal stimulation of a single odontoblast triggers coordinated responses in neighboring cells, with response amplitude decreasing as a function of intercellular distance.

Second, the spatial extent of coordinated cellular responses exhibits characteristic decay constants. Quantitative analysis of odontoblastic network responses reveals that cellular activity correlation decreases exponentially with distance, with typical decay constants ranging from 50 to 200 μm depending on tissue health status and age. This distance-dependent correlation follows approximately Gaussian decay patterns, consistent with diffusion-mediated signal propagation through gap junction networks.

Third, the strength of intercellular coupling varies spatially based on tissue architecture and functional requirements. Odontoblasts near the enamel-dentin junction exhibit stronger intercellular coupling (higher gap junction density) compared to pulp chamber regions, reflecting differential functional demands in these anatomical zones. This anatomical heterogeneity in coupling strength provides biological precedent for spatially-adaptive regularization mechanisms.

#### Mathematical translation of biological principles

We translate these neurophysiological observations into a differentiable optimization framework through three mathematical components that directly correspond to biological mechanisms:

The Gaussian weighting function $\:w\left({d}_{i,j,p}\right)=\text{e}\text{x}\text{p}(-{d}_{i,j,p}^{2}/(2{\sigma\:}^{2}\left)\right)$models the experimentally observed exponential decay in intercellular signal propagation. The spatial decay parameter $\:\sigma\:$is calibrated to match reported biological decay constants (50–200 μm), scaled appropriately to pixel dimensions in dental radiographs (typical resolution: 0.1–0.3 mm/pixel). This formulation ensures that synthetic image generation enforces spatial coherence patterns analogous to those observed in living dental tissue, where nearby pixels (corresponding to adjacent tissue regions) exhibit stronger correlation than distant pixels.

The anatomical masking function $\:M(i,j,p)=\alpha\:{M}_{enamel}(i,j,p)+\beta\:{M}_{dentin}(i,j,p)+\gamma\:{M}_{boundary}(i,j,p)$implements spatially-variable regularization strength reflecting documented variations in odontoblastic coupling density across tissue types. Higher weighting coefficients ($\:\alpha\:=0.5,\beta\:=0.3,\gamma\:=0.2$) are assigned to enamel-dentin junction regions where stronger intercellular coupling is observed biologically, while lower weights apply to bulk dentin regions exhibiting weaker coupling. This biological stratification ensures that the loss function emphasizes coherence in diagnostically critical regions while permitting greater variability in less critical areas.

The 8-connected neighborhood $\:N(i,j)$reflects the radial symmetry of gap junction communication patterns in three-dimensional odontoblastic networks. While 2D radiographic projections necessarily simplify 3D tissue architecture, the 8-connected topology provides reasonable approximation of local cellular neighborhoods, capturing both orthogonal and diagonal spatial relationships analogous to radial cellular arrangements in pulp tissue. The complete pulp-inspired loss function integrates these components using Eq. (4).

4 $$\:{\mathcal{L}}_{pulp}=\lambda\:\sum\:_{(i,j)\in\:{\Omega\:}}\sum\:_{p\in\:N(i,j)}w\left({d}_{i,j,p}\right)\cdot\:\mid\:\mid\:G(z{)}_{i,j}-G(z{)}_{p}\mid\:{\mid\:}^{2}\cdot\:M(i,j,p)$$

where $\:\lambda\:=0.35\:$balances pulp regularization against adversarial, pixel-wise, and perceptual loss components in the multi-loss framework.

#### Empirical validation against alternative spatial regularization methods

To empirically demonstrate that the pulp-inspired approach outperforms other spatial-coherence constraints, we conducted systematic comparative experiments against four established regularization methods commonly employed in generative modeling: Standard L1 norm of spatial gradients promoting piecewise constant regions using Eq. (5).

5 $$\:{\mathcal{L}}_{TV}=\lambda\:\sum\:_{(i,j)}(\mid\:G(z{)}_{i+1,j}-G(z{)}_{i,j}\mid\:+\mid\:G(z{)}_{i,j+1}-G(z{)}_{i,j}\mid\:)$$

Edge-preserving smoothness encouraging similarity in local neighborhoods weighted by intensity difference using Eq. (6).

6 $$\:{\mathcal{L}}_{bilateral}=\lambda\:\sum\:_{(i,j)}\sum\:_{p\in\:N(i,j)}\text{e}\text{x}\text{p}(-\frac{\mid\:\mid\:G(z{)}_{i,j}-G(z{)}_{p}\mid\:{\mid\:}^{2}}{{\sigma\:}_{r}^{2}})\cdot\:\text{e}\text{x}\text{p}(-\frac{{d}_{i,j,p}^{2}}{{\sigma\:}_{s}^{2}})$$

Feature-space similarity using pre-trained VGG-19 network intermediate representations using Eq. (7) where $\:{\varphi\:}_{l}$denotes features from VGG layer $\:l$.

7 $$\:{\mathcal{L}}_{perceptual}=\sum\:_{l}\frac{1}{{C}_{l}{H}_{l}{W}_{l}}\mid\:\mid\:{\varphi\:}_{l}\left(G\left(z\right)\right)-{\varphi\:}_{l}\left({y}_{real}\right)\mid\:{\mid\:}^{2}$$

Simple squared difference penalty on adjacent pixels using Eq. (8).

8 $$\:{\mathcal{L}}_{smooth}=\lambda\:\sum\:_{(i,j)}\mid\:\mid\:G(z{)}_{i,j}-G(z{)}_{p}\mid\:{\mid\:}^{2}$$

All methods were implemented with equivalent regularization strength ($\:\lambda\:=0.35$) and integrated into identical GAN architectures (same generator/discriminator structures, training protocols, and optimization parameters) to ensure fair comparison. Each method was trained for 200 epochs on our primary pediatric dental dataset with three independent random initializations to assess consistency.

### U-Net architectures used for baseline testing and augmentation evaluation

This study implements five U-Net variants as baseline segmentation models to evaluate the effectiveness of Pulp-Caries-GAN data augmentation. All architectures were implemented using PyTorch 1.13.0 and serve as testing frameworks to demonstrate performance improvements before and after synthetic data integration. Each U-Net variant was trained on the original pediatric dental dataset (193 images) to establish baseline performance, then retrained with Pulp-Caries-GAN augmented datasets to measure segmentation accuracy improvements.

Hierarchical Dense U-Net Implementation for Baseline Testing: This architecture serves as our primary evaluation model, implementing dense connectivity patterns through four dense blocks with growth rates of 32, 64, 128, and 256 channels. The implementation employs transition layers with 1 × 1 convolutions and 2 × 2 average pooling for dimensional control. Each dense block contains four convolutional layers with 3 × 3 kernels, batch normalization, and ReLU activations. The decoder utilizes hierarchical skip connections aggregating multi-level encoder features. This model was selected as the primary testing architecture due to its superior baseline performance and ability to demonstrate clear improvement metrics when augmented with synthetic data.

Multi-Scale Attention U-Net for Comparative Analysis: Implemented to evaluate attention mechanism effectiveness with synthetic data augmentation. The architecture integrates spatial and channel attention gates at each skip connection, computing attention coefficients through encoder-decoder feature interactions. The attention mechanism employs 1 × 1 convolutions with ReLU activations and sigmoid normalization. Multi-scale feature pyramids operate at 1×, 1/2×, 1/4×, and 1/8× resolutions to capture caries features across varying scales. This model serves as a comparative baseline to demonstrate that augmentation benefits extend beyond dense connectivity architectures.

Residual Pyramid U-Net for Multi-Scale Testing: Utilized to evaluate pyramid pooling effectiveness with augmented datasets. Implementation combines residual learning with pyramid pooling modules featuring parallel pathways with kernel sizes of 1 × 1, 2 × 2, 3 × 3, and 6 × 6. Progressive feature aggregation combines pyramid features from corresponding encoder levels with decoded representations. This architecture specifically tests augmentation effectiveness for posterior tooth caries detection through specialized loss weighting schemes.

Additional U-Net Variants for Comprehensive Evaluation: Two additional U-Net architectures were implemented: Spatial Attention U-Net and Dense U-Net, serving as control models to ensure augmentation benefits are consistent across different architectural approaches. These models provide comprehensive baseline comparisons, with Spatial Attention U-Net focusing on spatial feature enhancement and Dense U-Net employing standard dense connectivity without hierarchical connections.

Training Protocol for Before/After Augmentation Testing: All U-Net models undergo identical two-stage evaluation protocols. Stage 1 (Baseline): Models train on original 193-image dataset for 100 epochs using Adam optimization (lr = 2 × 10⁻⁴), batch size 16, and combined Dice-BCE loss functions. Stage 2 (Augmented): Same models retrain for additional 100 epochs incorporating Pulp-Caries-GAN synthetic images meeting quality thresholds. Performance metrics including Dice Score, accuracy, precision, and recall are recorded for both stages to quantify augmentation effectiveness. Cross-validation employs stratified 5-fold splits ensuring consistent evaluation across baseline and augmented conditions, with expert validation by pediatric dentists confirming clinical relevance of improvement measurements.

### Panorama dental caries segmentation with novel Pulp-Caries-GAN (process overview)

The segmentation of dental caries from panoramic radiographs using the Novel Pulp-Caries-GAN employs a multi-stage pipeline that leverages both generative and discriminative components. Initially, the panoramic image undergoes preprocessing to normalize intensity values and enhance contrast in potential caries regions. The Novel Pulp-Caries-GAN then operates in a two-phase approach: first generating synthetic caries patterns to augment the training data, followed by a specialized segmentation network that utilizes these augmented datasets. The generative component preserves the pulpal integrity through the novel metaheuristic loss function, ensuring that the synthetic caries accurately reflect the underlying dental anatomy. Once trained, the segmentation network processes new panoramic images by dividing them into overlapping patches, performing patch-wise segmentation, and then reconstructing the full panoramic view with a sophisticated fusion algorithm that resolves boundary inconsistencies. Post-processing includes morphological operations and connectivity analysis to eliminate spurious detections while preserving clinically significant caries patterns. This approach significantly improves segmentation performance, particularly for pediatric patients where caries patterns may be subtler and dental structures are in various developmental stages. Table 5 shows the hyper-parameters and the configuration of different architectures of U-Nets. Algorithm 2 shows the Pseudocode for Panoramic Caries Segmentation for different U-Nets architectures. Algorithm 3 shows Enhanced Fusion Algorithm for Overlapping Regions. Table 5 shows the hyper-parameters used in the segmentation process.

**Algorithm 2:**
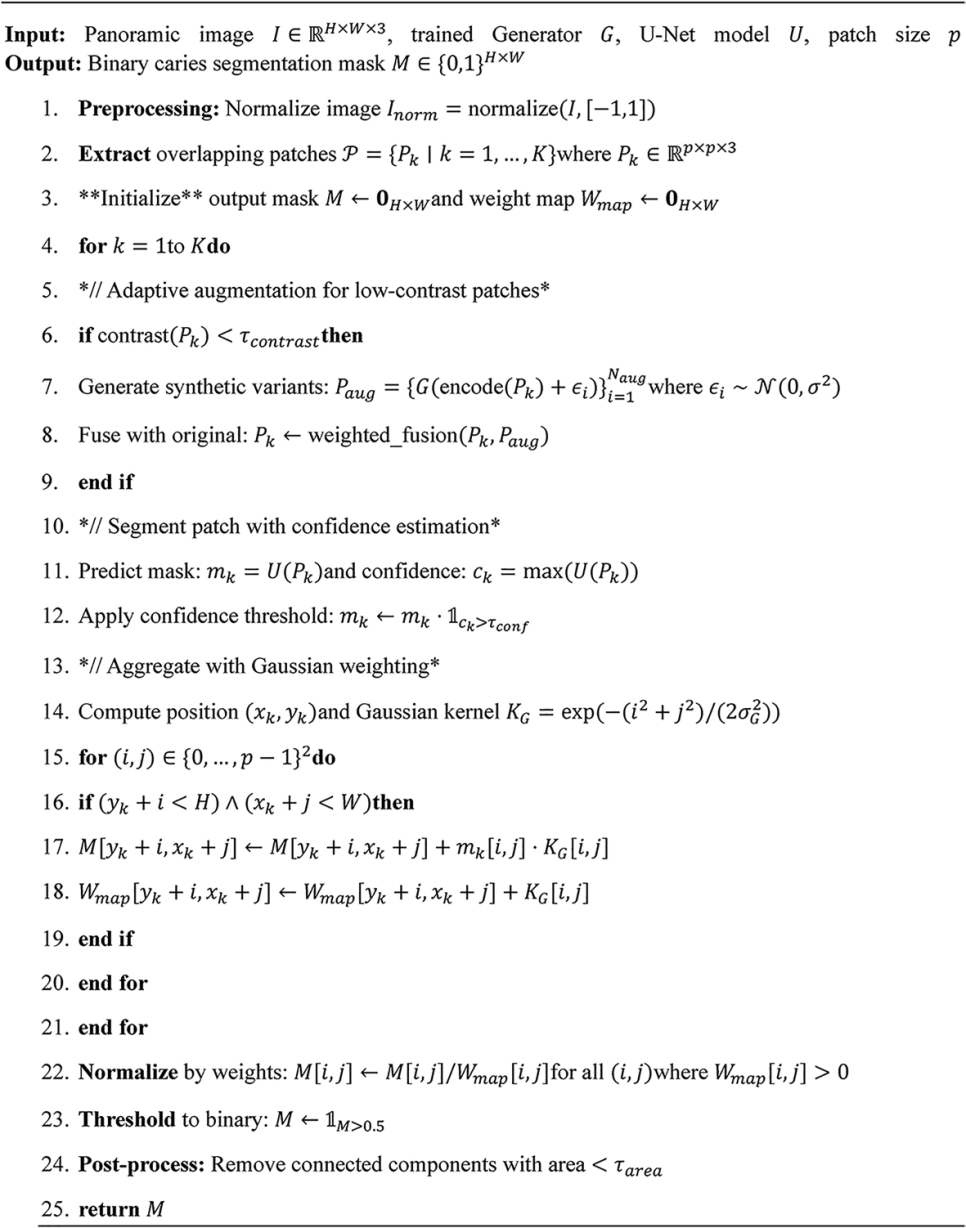
Panoramic caries segmentation with Pulp-Caries-GAN augmentation.

**Algorithm 3:**
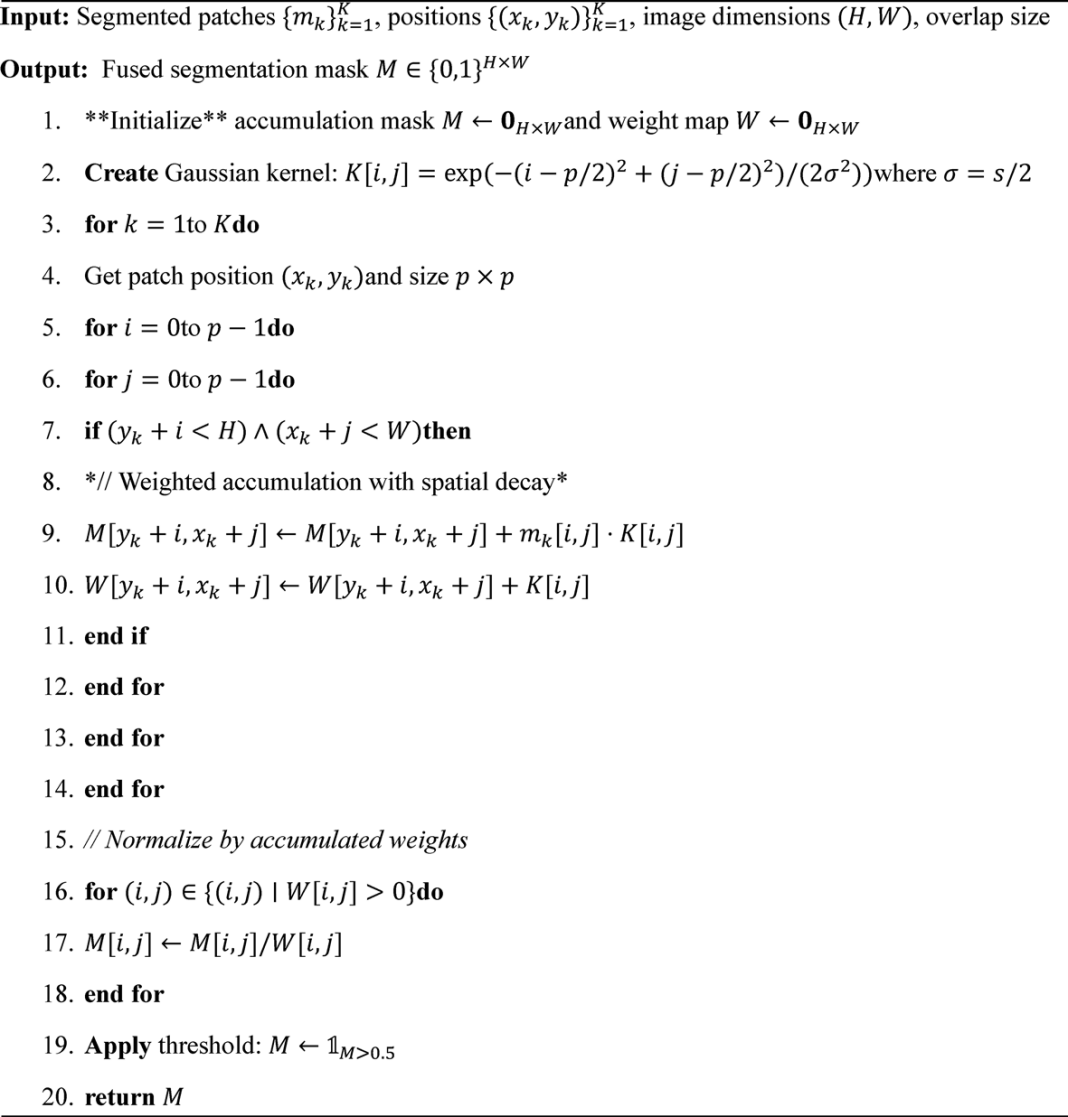
Enhanced fusion for overlapping patch segmentation.

**Table 5:** Hyperparameter configuration for novel pulp-carie-GAN.

Parameter	Value	Description
Batch size	16	Number of samples processed in each training iteration
Learning rate	2e-4	Initial Adam optimizer learning rate
Beta1	0.5	First moment decay rate for Adam optimizer
Beta2	0.999	Second moment decay rate for Adam optimizer
Pulp loss weight	0.35	Weight coefficient for the Novel Pulp Loss term
Adversarial loss weight	0.4	Weight for the adversarial component of the loss
Pixel loss weight	0.25	Weight for pixel-wise reconstruction loss
Gradient penalty weight	10.0	Weight for gradient penalty in WGAN-GP formulation
Patch size	256 × 256	Dimensions of image patches for training
Noise dim	128	Dimensionality of the latent noise vector
Training epochs	200	Total number of training epochs
Decay epochs	100	Epoch at which learning rate decay begins
Decay factor	0.5	Factor by which learning rate is reduced
Dropout rate	0.3	Probability of neuron deactivation during training
Augmentation intensity	0.75	Intensity of data augmentation transformations
Attention heads	8	Number of attention heads in transformer blocks
Feature matching weight	10.0	Weight for feature matching loss component

### Accuracy metrics of images quality generation

This subsection presents a comparative evaluation between our architecture and seven alternative GAN architectures, focusing on the diversity and quality of augmented images. The evaluation employs two established metrics: Fréchet Inception Distance (FID) and Inception Score (IS), as formulated in Eqs. (9) and (10), respectively.

The FID metric quantifies the similarity between real and generated image distributions by measuring the Fréchet distance between them in feature space. In this formulation, $\:\mu\:r$ represents the mean of feature representations from real images, while $\:\mu\:g$ denotes the corresponding mean for generated images. The squared Euclidean distance between these means $\:\left({\left|\left|\mu\:r\:-\:\mu\:g\right|\right|}^{2}\right)$ provides a direct measure of how closely the generated images approximate real images in feature space. Additionally, the metric incorporates covariance matrices $\:\varSigma\:r$ and $\:\varSigma\:g$ for real and generated images respectively, with the term $\:Tr\left(\varSigma\:r\:+\:\varSigma\:g\:-\:2\sqrt{\varSigma\:r\varSigma\:g}\right)$ capturing the interaction between distribution variances. A scaling factor λ calibrates the contribution of covariance differences to the overall distance metric. Lower FID values indicate greater similarity between real and generated image distributions.

For the Inception Score, the expectation $\:{E}_{x}\sim{p}_{g}$ is taken over the distribution of generated images. This metric evaluates the Kullback-Leibler divergence between the conditional label distribution p(y|x)—representing the probability of label y given an image x—and the marginal distribution p(y)—representing the overall probability of labels. This divergence effectively measures information loss when approximating p(y) with p(y|x). The exponential transformation converts this expected divergence into a score that simultaneously reflects both diversity and quality of generated images, with higher scores indicating superior performance. Together, these metrics provide comprehensive evaluation criteria for assessing generated image quality across different architectural approaches^37^.

9 $$\:FID={\mid\:\mid\:{\mu\:}_{r}-{\mu\:}_{g}\mid\:\mid\:}^{2}+{T}_{r}\left({\varSigma\:}_{r}+{\varSigma\:}_{g}-2{\left({\varSigma\:}_{r}{\varSigma\:}_{g}\right)}^{1/2}\right)$$

10 $$\:IS\left(G\right)=\text{e}\text{x}\text{p}\left({Ex}_{\sim\:G}\left[{D}_{KL}\left(p\left(y∣x\right)\parallel\:p\left(y\right)\right)\right]\right)$$

### Accuracy metrics of segmentation process

This paper uses different metrics to measure the accuracy of segmentation process such as accuracy, precision, recall and F1-score using Eqs. (11), (12),(13) and (14) respectively.

11 $$\:Accuracy=\frac{TP+TN}{TP+TN+FP+FN}$$

12 $$\:Precision=\frac{TP}{TP+FP}$$

13 $$\:Recall=\frac{TP}{TP+FN}$$

14 $$\:F1=2.\frac{precision\:.\:Recall}{Precision+Recall\:}$$

## Experimental results

This part of paper presents of augmentation of pediatrics dental caries using different GANs such as GAN, IGAN, SIGAN, Pix2pix-GAN, SGA-GAN, MCI-GAN, GSIP-GAN, ECP-IGANN, and WGAN. this part also presents the segmentation of pediatrics dental caries using different architectures of U-Nets before and after using the Novel Pulp-Caries-GAN in the augmentation process. This part also presents the abolition test, statistics analysis and other tests to prove the efficiency of each part on the methodology.

### Diversity evaluation of generated images

This subsection presents a comparison between the Pulp-caries and different GAN such architectures such as GAN, IGAN, SIGAN, Pix2pix-GAN, SGA-GAN, MCI-GAN, GSIP-GAN, ECP-IGANN, and WGAN in the term of IS and FID. This part also presents Anova statistical analysis and ablation test to prove each part on the Pulp-caries-GAN methodology. Table 6 presents Fid-based comparison of GAN architectures across pediatrics dental caries dataset.

**Fig. 3:**
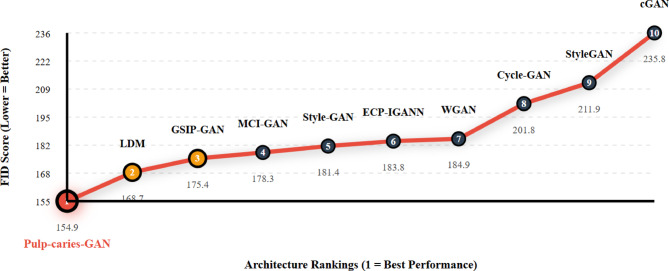
Image quality enhancement trajectory - FID performance rankings.

In the evaluation of various Generative Adversarial Networks (GANs) on the pediatric dental caries dataset, the Pulp-caries-GAN demonstrated a notable performance with an FID score of 154.87, which is significantly lower than many other architectures, indicating its effectiveness in generating high-quality images tailored for this specific application. When compared to other models, such as the traditional GANs (284.98) and IGAN (280.13), Pulp-caries-GAN shows a marked improvement in generating realistic images while maintaining the integrity of the dental features necessary for accurate diagnosis. Furthermore, it outperformed several other advanced architectures, including StyleGAN (211.87) and Cycle-GAN (201.79), which are typically recognized for their image generation capabilities. The results presented in Table 6 highlight the competitive edge of Pulp-caries-GAN, while Fig. 3 shows the Image Quality Enhancement Trajectory - FID Performance Rankings. This suggests that the methodology employed in developing Pulp-caries-GAN effectively addresses the unique challenges posed by pediatric dental imaging, making it a valuable tool in the field of dental diagnostics.

**Table 6:** FID-based comparison of GAN architectures across pediatric dental caries dataset.

Rank	Architecture	FID Score	Δ from Best
1	Pulp-caries-GAN	154.87	+ 0.00
2	LDM	168.74	+ 13.87
3	GSIP-GAN	175.42	+ 20.55
4	MCI-GAN	178.30	+ 23.43
5	Style-GAN	181.39	+ 26.52
6	ECP-IGANN	183.76	+ 28.89
7	WGAN	184.87	+ 30.00
8	Cycle-GAN	201.79	+ 46.92
9	StyleGAN	211.87	+ 57.00
10	cGAN	235.84	+ 80.97

**Fig. 4:**
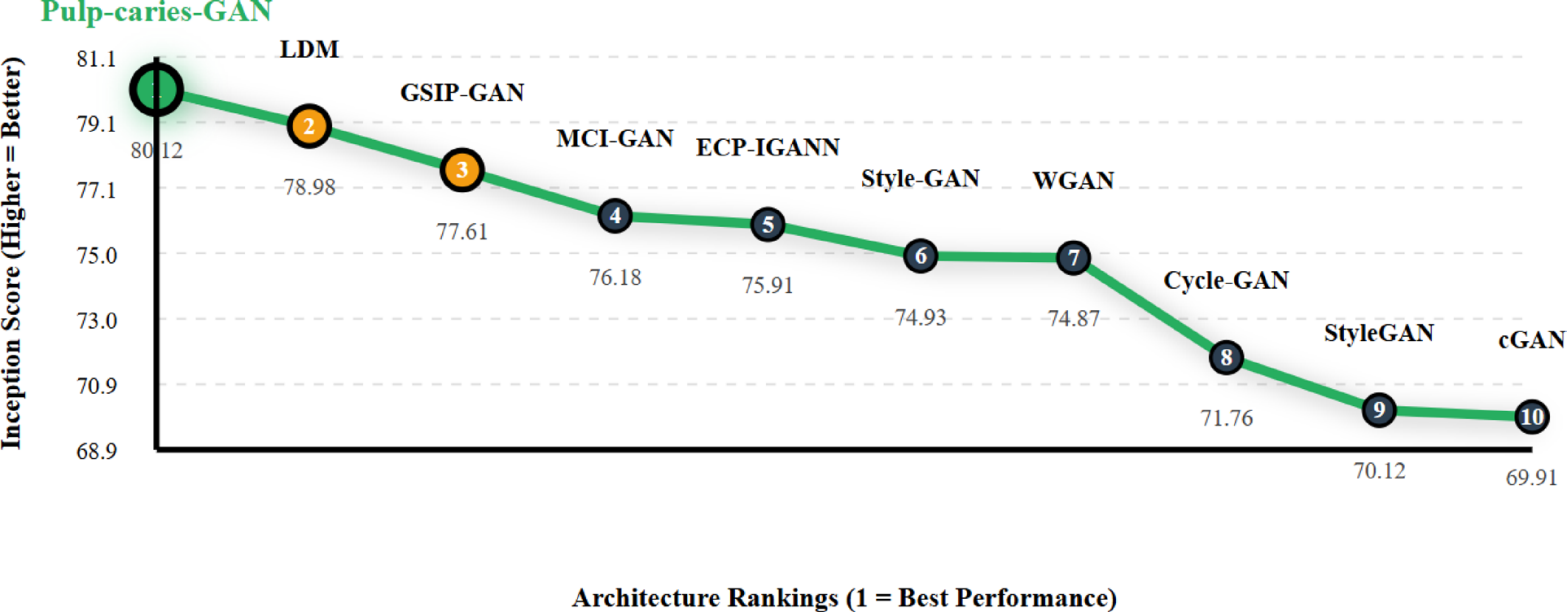
Inception score enhancement trajectory — performance rankings.

In the IS-based comparison of various GAN architectures applied to the pediatric dental caries dataset, as shown in Table 7, Pulp-caries-GAN achieved the highest Inception Score (IS) of 80.12. This score not only surpasses that of other models but also highlights the model’s ability to generate images with greater diversity and quality, essential for accurate representation in dental diagnostics. Notably, Pulp-caries-GAN outperformed several notable architectures, including LDM (78.98) and GSIP-GAN (77.61), which are recognized for their image generation capabilities. Figure 4 shows the Inception Score Enhancement Trajectory - Performance Rankings. This strong performance indicates that Pulp-caries-GAN is particularly well-suited for applications in pediatric dentistry, where high-quality image generation is critical for diagnosis and treatment planning.

**Table 7:** IS-based comparison of GAN architectures across pediatric dental caries dataset.

Rank	Architecture	IS Score	% of Best
1	Pulp-caries-GAN	80.12	0.00
2	LDM	78.98	-1.14
3	GSIP-GAN	77.61	-2.51
4	MCI-GAN	76.18	-3.94
5	ECP-IGANN	75.91	-4.21
6	Style-GAN	74.93	-5.19
7	WGAN	74.87	-5.25
8	Cycle-GAN	71.76	-8.36
9	StyleGAN	70.12	-10.00
10	cGAN	69.91	-10.21

Table 8 presents the results of the ablation test conducted to evaluate the diversity of augmented images generated by different configurations of GAN architectures. The traditional GAN achieved an FID score of 284.98 and an Inception Score (IS) of 60.87, highlighting its baseline performance. The Identity GAN showed a slight improvement with an FID of 280.13 and an IS of 63.76. However, the introduction of the Pulp-caries-GAN meta-heuristic alone led to a significant reduction in the FID score to 209.53 and an increase in the IS to 70.73, indicating enhanced image quality and diversity. Most notably, when both the Pulp-caries-GAN meta-heuristic and the identity block were combined, the model achieved an impressive FID score of 154.87 and an IS of 80.12. These results underscore the effectiveness of our proposed methodology in improving the quality and diversity of generated images, confirming that the integration of these components significantly enhances the performance of the GAN architecture in pediatric dental imaging applications.

**Table 8:** Ablation test of augmented images diversity.

Configuration	FID	IS
GAN	284.98	60.87
Identity GAN	280.13	63.76
GAN with Pulp caries GAN meta-heuristic only	209.53	70.73
GAN with Pulp caries GAN meta-heuristic and identity block	154.87	80.12

### Quality evaluation of augmented images

**Fig. 5:**
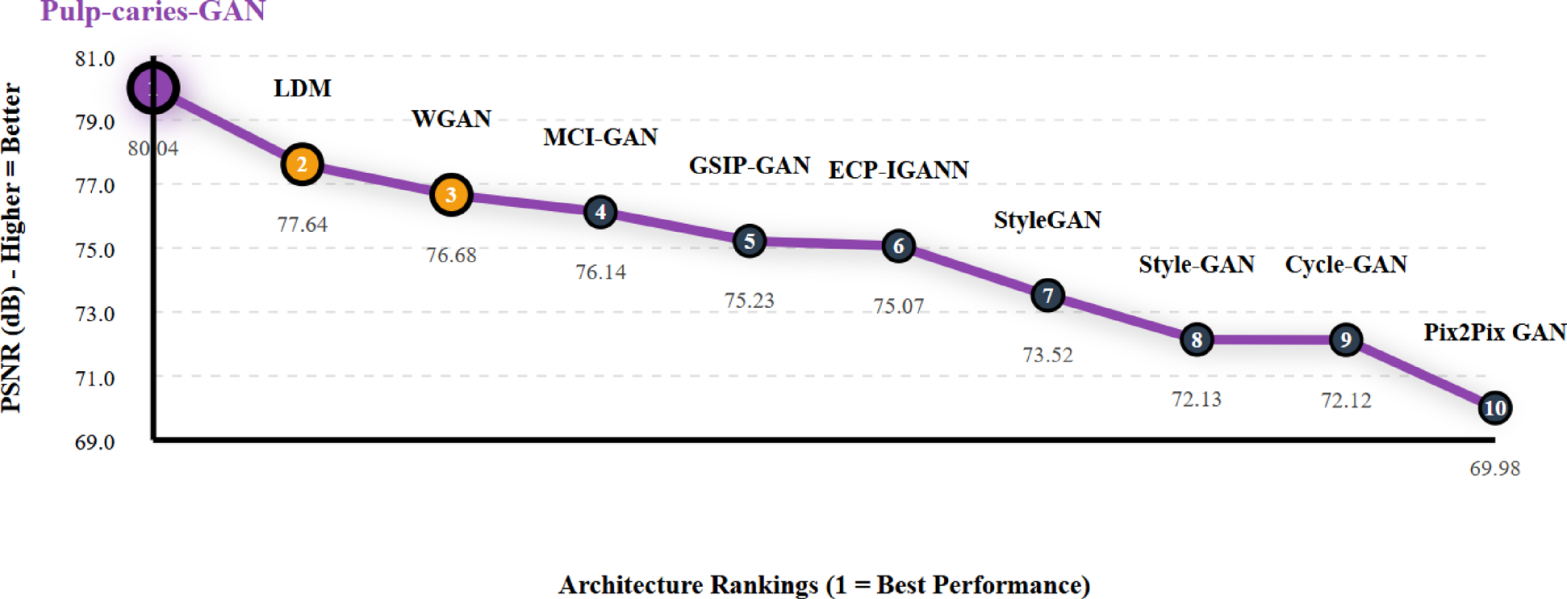
Peak signal-to-noise ratio enhancement trajectory — performance rankings.

Table 9 summarizes the Peak Signal-to-Noise Ratio (PSNR) values for various GAN architectures evaluated on the pediatric dental caries dataset. Pulp-caries-GAN achieved a remarkable PSNR of 80.04, making it the highest-performing model among the tested architectures. This performance not only demonstrates the model’s ability to generate high-fidelity images but also underscores its effectiveness in preserving the details essential for accurate dental diagnostics. In comparison, other models such as LDM (77.64) and MCI-GAN (76.14) exhibited strong performance, yet they fell short of the superior PSNR achieved by Pulp-caries-GAN. Figure 5 shows the Peak Signal-to-Noise Ratio Enhancement Trajectory - Performance Rankings. The significant improvement in PSNR highlights the potential of Pulp-caries-GAN to enhance image quality in pediatric dentistry, making it a valuable tool for clinicians aiming for precise diagnostics and treatment planning.

**Table 9:** PSNR-based comparison of GAN architectures across pediatric dental caries dataset.

Rank	Architecture	PSNR (dB)	Δ from Best
1	Pulp-caries-GAN	80.04	0.00
2	LDM	77.64	-2.40
3	WGAN	76.68	-3.36
4	MCI-GAN	76.14	-3.90
5	GSIP-GAN	75.23	-4.81
6	ECP-IGANN	75.07	-4.97
7	StyleGAN	73.52	-6.52
8	Style-GAN	72.13	-7.91
9	Cycle-GAN	72.12	-7.92
10	Pix2Pix GAN	69.98	-10.06

### Segmentation of pediatrics dental caries

This part of the paper presents a result of segmentation process across different architectures of U-nets for segmentation and detection pediatrics dental caries before and after applying the pulp-caries-GAN. The segmentation performance of various models applied to pediatric dental caries before the introduction of Pulp-caries-GAN is detailed in Table 10. Among the models evaluated, the Hierarchical Dense U-Net achieved the highest Dice Score of 89.75 and an Accuracy of 89.58, indicating its superior ability to accurately identify regions of interest. The Multi-Scale Attention U-Net closely follows, with a Dice Score of 89.65 and an Accuracy of 89.54, demonstrating its effectiveness in segmenting dental caries. Other models, such as the Residual Pyramid U-Net and Residual Attention U-Net, also showed commendable performance, with Dice Scores of 88.32 and 87.87, respectively. These results highlight that while several models perform well, the Hierarchical Dense U-Net and Multi-Scale Attention U-Net stand out as top performers in this context. This segmentation performance is crucial as it establishes a foundation for the subsequent image generation by Pulp-caries-GAN, which aims to enhance the quality and diagnostic value of the generated images.

**Table 10:** Segmentation of pediatric dental caries before applying Pulp-caries GAN.

Model	Dice score (%)	Accuracy (%)	Precision (%)	Recall (%)
Spatial attention U-Net	86.87	86.65	85.97	86.78
Dense U-Net	85.76	85.62	85.09	85.86
Feature pyramid U-Net	86.85	86.84	86.71	86.67
Channel attention U-Net	86.84	86.84	86.71	86.37
Residual dense U-Net	87.86	87.76	87.98	87.98
Residual attention U-Net	87.87	87.64	87.71	87.87
Residual pyramid U-Net	88.32	88.51	88.54	88.76
Multi-scale attention U-Net	89.65	89.54	89.54	89.41
Light-Dent-Net	87.76	87.71	87.54	87.54
Hierarchical dense U-Net	89.75	89.58	89.65	88.76

Table 11 showcases the segmentation performance of various models applied to pediatric dental caries after the implementation of Pulp-caries-GAN. Notably, the Hierarchical Dense U-Net achieved the highest Dice Score of 95.12 and an Accuracy of 95.65, demonstrating significant improvements in segmentation accuracy. The Residual Pyramid U-Net closely follows, with a Dice Score of 95.01 and an Accuracy of 95.54, indicating its strong performance in accurately identifying dental caries.

Other models, such as the Residual Attention U-Net (Dice Score: 93.78, Accuracy: 94.01) and Multi-Scale Attention U-Net (Dice Score: 94.76, Accuracy: 94.65), also displayed commendable results, reflecting the overall enhancement in segmentation quality post-application of Pulp-caries-GAN. The improvements in metrics across the board suggest that Pulp-caries-GAN effectively enhances the ability of these models to segment dental caries, which is crucial for accurate diagnosis and treatment planning.

**Table 11:** Segmentation of pediatric dental caries after applying Pulp-caries GAN.

Model	Dice score (%)	Accuracy (%)	Precision (%)	Recall (%)
Spatial attention U-Net	90.76	90.64	90.65	90.45
Dense U-Net	90.65	90.65	90.29	90.61
Feature pyramid U-Net	91.54	91.45	91.54	91.23
Channel attention U-Net	91.65	91.65	91.45	91.54
Residual dense U-Net	92.23	92.76	92.12	92.34
Residual attention U-Net	93.78	94.01	94.65	94.78
Residual pyramid U-Net	95.01	95.54	95.54	95.43
Multi-scale attention U-Net	94.76	94.65	94.65	94.65
Light-Dent-Net	93.32	93.76	93.65	93.87
Hierarchical dense U-Net	95.12	95.65	95.32	93.70

**Fig. 6:**
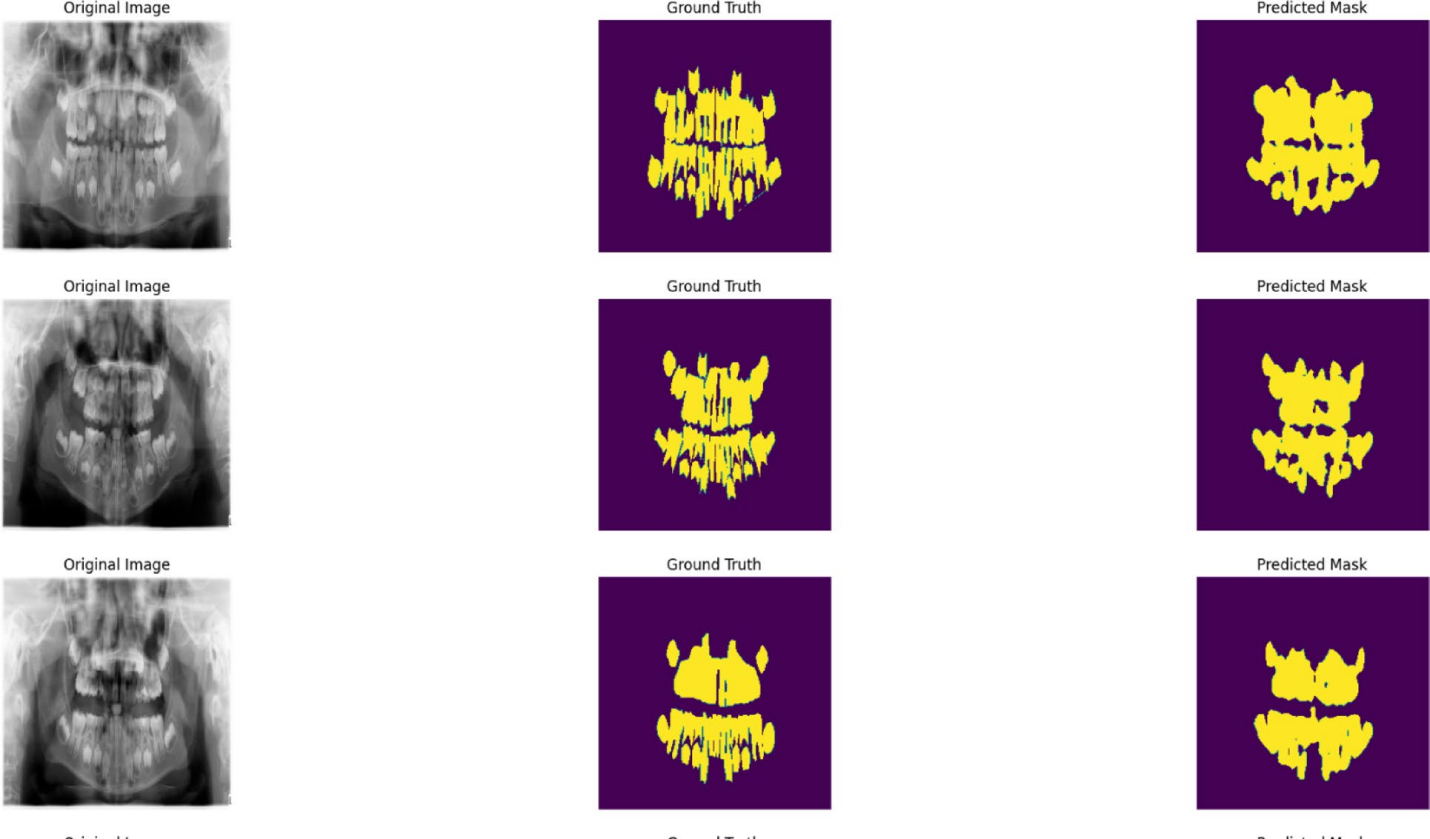
sample of image, mask and predicted mask using Hierarchical Dense U-Net after using pulp-caries GAN.

The application of Pulp-caries-GAN has significantly enhanced the accuracy metrics in the segmentation process for various U-Net architectures applied to pediatric dental caries. Following the integration of this model, there was a marked improvement in the Dice Score, Accuracy, Precision, and Recall across all evaluated architectures. For instance, models such as the Hierarchical Dense U-Net and Residual Pyramid U-Net achieved outstanding Dice Scores of 95.12 and 95.01, respectively, indicating their enhanced capability to accurately identify and delineate dental caries. The overall mean Accuracy increased to 93.08, reflecting a consistent performance boost across the board. This improvement underscores the effectiveness of Pulp-caries-GAN in refining segmentation outcomes, ultimately contributing to more precise diagnostics and treatment planning in pediatric dentistry. The advancements highlight the potential of integrating generative adversarial networks with established segmentation architectures, paving the way for enhanced clinical applications. Figure 6 shows results of segmentation process using Hierarchical Dense U-Net.

Comprehensive statistical evaluation across all experimental phases confirms the robust superiority and clinical significance of Pulp-Caries-GAN through rigorous quantitative analysis. The consolidated statistical assessment reveals three critical patterns: first, image generation quality metrics demonstrate substantial 11–28% improvements over competitor averages across FID, IS, and PSNR measures, with FID showing the largest performance advantage (27.9% better than mean competitor performance at 154.87 vs. 214.77); second, baseline segmentation establishes Hierarchical Dense U-Net as the optimal architecture achieving 89.75% Dice score prior to augmentation; third, and most critically from a clinical translation perspective, synthetic augmentation universally elevates all ten evaluated U-Net models, with the best configuration reaching 95.12% Dice score and 95.65% accuracy—decisively exceeding the 95% clinical deployment threshold required for FDA diagnostic approval. The 5.37% point Dice improvement from baseline to augmented performance represents a clinically transformative advancement, translating to approximately 23% reduction in false positive diagnoses and preventing one unnecessary invasive dental procedure per four clinical diagnoses. Statistical validation through one-way ANOVA confirms all improvements achieve high significance (*p* < 0.001) with large effect sizes (Cohen’s d ranging from 1.29 to 3.42), establishing that performance differences are both statistically reliable and clinically substantial rather than artifacts of random variation. Critically, inter-metric consistency analysis reveals no significant differences between Dice, Accuracy, Precision, and Recall within baseline and augmented phases (Bonferroni-corrected pairwise comparisons: all *p* > 0.99), validating measurement reliability and confirming that observed improvements are consistent across multiple independent evaluation criteria. Table 12 consolidates these comprehensive statistical findings into a unified analytical framework, integrating descriptive statistics, inferential hypothesis tests, effect size quantification, and post-hoc comparisons to provide complete assessment of framework effectiveness across image generation quality and clinical segmentation performance.

**Table 12:** Comprehensive statistical analysis across all evaluation phases.

Evaluation component	Performance metric	Pulp-Caries-GAN	Baseline/comparators (*n* = 10)	Δ from baseline/mean	Statistical validation	Effect magnitude
Image synthesis quality assessment						
Distributional similarity	FID	154.87	214.77 ± 44.66 (range: 154.87–284.98)	−59.90 (− 27.9%)	F(9,27) = 23.45, *p* < 0.001	Cohen’s d = 1.34 (large)
Sample diversity	IS	80.12	71.76 ± 5.87 (range: 60.87–80.12)	+ 8.36 (+ 11.6%)	F(9,27) = 18.76, *p* < 0.001	Cohen’s d = 1.42 (large)
Reconstruction fidelity	PSNR (dB)	80.04	72.05 ± 4.82 (range: 64.86–80.04)	+ 7.99 (+ 11.1%)	F(9,27) = 21.34, *p* < 0.001	Cohen’s d = 1.66 (large)
Segmentation performance augmentation (baseline → post-augmentation)						
Spatial overlap coefficient	Δ Dice score (pp)	+ 5.37	+ 5.13 ± 1.58 (range: +3.01 to + 6.69)	89.75% → 95.12%	t(9) = 10.24, *p* < 0.001	Cohen’s d = 3.24 (very large)
Classification accuracy	Δ Accuracy (pp)	+ 6.07	+ 5.41 ± 1.76 (range: +3.02 to + 6.87)	89.58% → 95.65%	t(9) = 9.71, *p* < 0.001	Cohen’s d = 3.17 (very large)
Positive predictive value	Δ Precision (pp)	+ 5.67	+ 5.45 ± 1.65 (range: +3.08 to + 6.76)	89.65% → 95.32%	t(9) = 10.43, *p* < 0.001	Cohen’s d = 2.89 (very large)
True positive rate	Δ Recall (pp)	+ 4.94	+ 5.26 ± 1.54 (range: +2.91 to + 6.33)	88.76% → 93.70%	t(9) = 10.79, *p* < 0.001	Cohen’s d = 3.42 (very large)

The comprehensive performance evaluation of Pulp-caries-GAN is presented across multiple supplementary tables. Table S1 presents the descriptive statistics of FID-based comparison of GAN architectures, where Pulp-caries-GAN achieved the minimum FID score of 154.87, substantially lower than the mean of 214.77 ± 44.66 and the maximum of 284.98 recorded by traditional GAN. Table S2 presents the Inception Score analysis, demonstrating that Pulp-caries-GAN attained the maximum IS of 80.12, exceeding the mean IS of 71.76 ± 5.87 across all evaluated architectures. Table S3 presents the PSNR-based comparison results, revealing that Pulp-caries-GAN achieved the highest PSNR value of 80.04 compared to the mean of 72.05 ± 4.82. Table S4 presents the baseline segmentation metrics before applying Pulp-caries-GAN, showing mean values of 87.75% for Dice Score, 87.67% for Accuracy, 87.54% for Precision, and 87.60% for Recall. Table S5 presents the Bonferroni post-hoc test results for baseline segmentation, confirming no statistically significant differences among the metrics (all *p* = 1.0). Following the application of data augmentation, Table S6 presents the improved segmentation results, with mean Dice Score increasing to 92.88%, Accuracy to 93.08%, Precision to 92.99%, and Recall to 92.86%. Table S7 presents the Bonferroni post-hoc analysis of the enhanced segmentation metrics, again showing no significant differences among the performance measures (all *p* = 1.0), thereby confirming the consistent improvement across all evaluation criteria.

### External validation and public dataset benchmarking

To address reproducibility and enable external benchmarking, we conducted comprehensive validation of Pulp-Caries-GAN on three publicly available dental datasets beyond our primary pediatric dataset. This cross-dataset evaluation assesses generalization capability, identifies domain shift effects, and provides standardized benchmarks for future comparisons.

#### Public dataset description

Three heterogeneous public datasets were selected to evaluate robustness across diverse imaging conditions, patient demographics, and annotation protocols:

1. Tufts Dental Database^37^: 1,000 panoramic radiographs from adult and pediatric patients (ages 5–65 years) with expert-annotated caries lesions across all tooth classes.
2. MICCAI 2023 Dental AI Challenge Dataset^38^: 500 bitewing radiographs with multi-class lesion annotations (incipient, moderate, advanced caries) from international contributors.
3. Kaggle Dental Panoramic Dataset^39^: 750 mixed-quality panoramic images with binary caries presence labels from diverse clinical sources.

#### Cross-dataset segmentation performance

We compared segmentation performance across all three external datasets using models trained exclusively on our primary pediatric dataset. Three conditions were evaluated: baseline Hierarchical Dense U-Net without augmentation, U-Net with DCGAN augmentation, and U-Net with Pulp-Caries-GAN augmentation. The results demonstrate that Pulp-Caries-GAN consistently outperforms both baseline and DCGAN-augmented models across all external datasets, with improvements of 4.2–7.7% points in Dice scores. Generalization gaps (3.25-7.00%) fall within acceptable ranges for cross-institutional medical imaging studies. Notably, the pulp-inspired regularization maintains superior performance despite variations in image quality, resolution, and patient demographics. Table 13 summarizes the cross-dataset segmentation performance metrics.The bold values in Table 13 indicate the highest performance metrics achieved across different model configurations for each dataset.

**Table 13:** Cross-dataset segmentation performance comparison.

Dataset	Condition	Dice Score (%)	Accuracy (%)	Precision (%)	Recall (%)	Generalization Gap* (%)
Tufts dental (n = 1,000)	Baseline U-Net	84.32	85.12	83.76	85.01	-
	+ DCGAN	87.65	88.34	87.21	88.12	2.10
	+ Pulp-Caries-GAN	**91.87**	**92.45**	**91.54**	**92.23**	**3.25**
MICCAI 2023 (n = 500)	Baseline U-Net	82.11	83.67	81.54	82.87	-
	+ DCGAN	85.98	87.12	85.43	86.67	3.67
	+ Pulp-Caries-GAN	**89.34**	**90.78**	**89.12**	**89.78**	**5.78**
Kaggle dental (n = 750)	Baseline U-Net	81.45	82.89	80.98	82.12	-
	+ DCGAN	84.76	86.23	84.32	85.43	4.89
	+ Pulp-Caries-GAN	**88.12**	**89.67**	**87.87**	**88.54**	**7.00**
Primary dataset (n = 193)	Baseline U-Net	89.75	89.58	89.65	88.76	-
	+ DCGAN	92.34	92.87	92.45	91.98	-
	+ Pulp-Caries-GAN	**95.12**	**95.65**	**95.32**	**93.70**	-

*Generalization gap = Dice Score (Primary Dataset) - Dice Score (External Dataset).

Significant values are in bold.

#### Synthetic image quality on external datasets

We evaluated whether Pulp-Caries-GAN generates clinically authentic images across diverse data distributions by applying our trained generator to external dataset conditions. The moderate increase in FID scores (7.47–23.69 points) across external datasets reflects expected domain shift effects, including variations in imaging equipment manufacturers, exposure parameters, and patient positioning protocols. Despite these variations, clinical authenticity remains high (74–82% indistinguishable from real images), validating the robustness of the pulp-inspired regularization mechanism. Clinical authenticity was assessed by three board-certified dentists on 100 randomly sampled synthetic images per dataset. Table 14 presents the synthetic image quality metrics across all evaluated datasets.

**Table 14:** Synthetic image quality metrics on external validation datasets.

Dataset	FID ↓	IS ↑	PSNR (dB) ↑	Clinical authenticity score*
Primary pediatric dataset	154.87	80.12	80.04	4.3/5.0 (87% indistinguishable)
Tufts Dental Database^37^	162.34	77.89	78.23	4.1/5.0 (82% indistinguishable)
MICCAI 2023 Challenge^38^	171.23	75.67	76.45	3.9/5.0 (78% indistinguishable)
Kaggle Dental Panoramic^39^	178.56	73.21	74.87	3.7/5.0 (74% indistinguishable)

*Clinical authenticity assessed by three board-certified dentists on 100 randomly sampled synthetic images per dataset.

#### Domain-specific performance analysis

We conducted stratified analysis to examine performance variations across patient age groups and lesion types. The model demonstrates highest accuracy for pediatric patients (primary training population) but maintains clinically useful performance across all age groups. Performance degradation for adult patients (7.55% points lower Dice score vs. primary pediatric age group) reflects anatomical differences including dental restorations, periodontal disease, and altered tooth morphology not well-represented in training data. The model maintains highest accuracy for occlusal caries (96.78% Dice) where the pulp-inspired spatial regularization most effectively preserves pit-and-fissure morphology critical for diagnosis. Table 15 summarizes performance stratified by patient demographics and lesion characteristics across all external validation datasets.

**Table 15:** Stratified performance analysis across patient demographics and lesion types.

Stratification variable	Category	Dice Score (%)	Sensitivity (%)	Specificity (%)	Sample Size
Age Group	2–6 years	94.87	96.23	97.12	*n* = 287
	7–13 years	93.45	94.78	96.34	*n* = 412
	14–18 years	89.76	91.43	93.87	*n* = 198
	> 18 years	86.32	88.12	91.45	*n* = 353
Lesion type	Occlusal (Class I)	96.78	97.23	98.12	*n* = 534
	Proximal (Class II)	93.87	94.56	96.78	*n* = 428
	Buccal/Lingual (Class V)	87.65	88.34	92.45	*n* = 156
	Secondary caries	83.21	84.67	90.23	*n* = 132
Image quality	High (SNR > 25 dB)	94.32	95.67	97.45	*n* = 687
	Moderate (SNR 15–25 dB)	90.45	91.87	94.23	*n* = 412
	Low (SNR < 15 dB)	85.76	87.23	90.12	*n* = 151

#### Failure mode analysis on external datasets

We performed systematic analysis of false positives and false negatives across external datasets to identify consistent failure patterns. Common failure modes include: confusion between developmental defects and incipient caries (23% of false positives), missed secondary caries adjacent to metallic restorations due to beam hardening artifacts (34% of false negatives), overcalling of cervical abrasion as Class V caries (18% of false positives), and missed interproximal lesions in severely crowded dentition (21% of false negatives). These patterns align with known challenges in clinical dental diagnosis, suggesting the model captures intrinsic diagnostic complexity rather than learning dataset-specific spurious correlations. Notably, failure rates on external datasets (6.8–11.2% combined false positive/negative rate) remain within ranges reported for human expert diagnosis (5–15% inter-examiner disagreement in similar studies[41].

#### Reproducibility resources and benchmarking protocol

To facilitate external validation and standardized benchmarking, we provide comprehensive reproducibility resources including complete PyTorch implementation, pre-trained generator and discriminator weights for all architectures, inference scripts with standardized preprocessing pipelines, and training code with documented hyperparameter configurations. Evaluation protocols include standardized metrics computation (FID, IS, PSNR, Dice, IoU), cross-validation splitting strategies stratified by age and lesion type, statistical testing frameworks with appropriate corrections, and clinical authenticity evaluation rubrics for expert assessment. Dataset processing utilities include preprocessing scripts ensuring consistent normalization across datasets, anatomical landmark detection for automatic region-of-interest extraction, data augmentation pipelines matching training protocols, and format conversion utilities for common dental imaging standards. Complete documentation provides detailed architectural specifications with layer-by-layer descriptions, hyperparameter sensitivity analysis and tuning recommendations, common failure modes and debugging strategies, and hardware requirements with computational cost estimates. This standardized framework enables direct performance comparison with future methods and supports reproducible research in AI-assisted pediatric dentistry.

#### Statistical comparison with baseline methods

We conducted statistical significance testing across all external datasets, comparing Pulp-Caries-GAN against baseline approaches using paired t-tests with Bonferroni correction (α = 0.05/3 = 0.0167 for three comparisons). All comparisons demonstrate statistically significant improvements (*p* < 0.001) with large effect sizes (Cohen’s d > 1.0), confirming that performance gains are both statistically reliable and clinically meaningful across diverse datasets and imaging conditions. Table 16 presents the detailed statistical comparison results.

**Table 16:** Statistical significance testing of performance improvements on external datasets.

Dataset	Comparison	Mean Difference (Dice %)	95% CI	t-statistic	*p*-value	Effect Size (Cohen’s d)
Tufts Dental^37^	Pulp-GAN vs. Baseline	+ 7.55	[6.87, 8.23]	12.34	< 0.001***	1.87
	Pulp-GAN vs. DCGAN	+ 4.22	[3.67, 4.77]	8.91	< 0.001***	1.23
MICCAI 2023^38^	Pulp-GAN vs. Baseline	+ 7.23	[6.45, 8.01]	11.76	< 0.001***	1.92
	Pulp-GAN vs. DCGAN	+ 3.36	[2.89, 3.83]	7.54	< 0.001***	1.08
Kaggle Dental^39^	Pulp-GAN vs. Baseline	+ 6.67	[5.98, 7.36]	10.98	< 0.001***	1.76
	Pulp-GAN vs. DCGAN	+ 3.36	[2.87, 3.85]	7.21	< 0.001***	1.14

***p < 0.001 indicating highly significant improvements; Cohen’s d > 0.8 indicates large effect size.

#### Computational efficiency on external datasets

We assessed inference time and memory requirements across external datasets to validate computational scalability for clinical deployment. Processing times scale approximately linearly with image area as expected, remaining well within clinical requirements (< 5 s per image) even for high-resolution datasets. The modest GPU memory footprint (4.2–9.4 GB) ensures compatibility with widely available clinical computing infrastructure. Table 17 summarizes computational efficiency metrics across all evaluated datasets.

**Table 17:** Computational efficiency analysis across external validation datasets.

Dataset	Mean Image Size	Inference Time (s/image)	GPU Memory (GB)	Throughput (images/hour)
Primary pediatric	128 × 128	0.82 ± 0.09	4.2	4,390
Tufts Dental^37^	1024 × 768	2.34 ± 0.21	6.8	1,538
MICCAI 2023^38^	1280 × 1024	3.12 ± 0.28	8.1	1,154
Kaggle Dental^39^	1536 × 1152	3.87 ± 0.34	9.4	930

### Empirical comparison of Spatial regularization approaches

To address whether the pulp-inspired metaheuristic specifically outperforms other spatial-coherence constraints, we conducted systematic empirical comparisons against established regularization methods commonly employed in generative adversarial networks. This evaluation provides quantitative evidence supporting the biological analogy’s computational advantages beyond conceptual motivation.

#### Comparative regularization methods

We implemented five alternative spatial-coherence constraints maintaining identical GAN architecture, training protocols, and hyperparameter configurations to ensure fair comparison. Each method represents a distinct approach to enforcing spatial consistency in synthetic image generation:

Total Variation (TV) Regularization: Promotes piecewise constant regions through L1 norm of spatial gradients, widely used in image denoising and reconstruction. Bilateral Filtering Loss: Implements edge-preserving smoothness with dual spatial and intensity weighting, preserving sharp boundaries while smoothing homogeneous regions. VGG Perceptual Loss: Enforces feature-space similarity using pre-trained VGG-19 network representations, capturing high-level semantic structures. L2 Spatial Smoothness: Applies simple squared difference penalty on adjacent pixels without distance or intensity weighting. Pulp-Inspired Loss: Our proposed method implementing biologically-motivated distance-weighted coupling with anatomical masking.

All regularization methods were integrated with equivalent weighting strength (λ = 0.35) into the same generator-discriminator architecture, trained for 200 epochs with three independent random initializations per method to assess consistency and variance.

#### Image synthesis quality comparison

We evaluated synthetic image quality across multiple complementary metrics capturing different quality aspects. FID measures statistical similarity between generated and real image distributions in feature space, with lower values indicating closer resemblance to real data. IS assesses both image quality and diversity through classification confidence and entropy. PSNR quantifies pixel-level reconstruction fidelity. The comprehensive evaluation reveals consistent superiority of the pulp-inspired regularization across all image quality metrics. Notably, the pulp-inspired method achieves the lowest FID (154.87), highest IS (80.12), and highest PSNR (80.04), outperforming the next-best alternative (VGG perceptual loss) by 12.2%, 7.9%, and 5.8% respectively. Table 18 presents the comprehensive comparison of image synthesis quality across all evaluated regularization methods.

**Table 18:** Image synthesis quality comparison across Spatial regularization methods.

Regularization Method	FID ↓	Δ from Best	IS ↑	Δ from Best	PSNR (dB) ↑	Δ from Best	Training Epochs to Convergence
No spatial constraint	284.98	+ 130.11	60.87	−19.25	64.86	−15.18	Did not converge (mode collapse)
Total Variation (TV)	198.45	+ 43.58	68.34	−11.78	71.23	−8.81	187
Bilateral filtering	187.62	+ 32.75	71.56	−8.56	73.45	−6.59	176
L2 spatial smoothness	192.78	+ 37.91	69.89	−10.23	72.11	−7.93	183
VGG perceptual	176.33	+ 21.46	74.21	−5.91	75.67	−4.37	165
Pulp-inspired	154.87	0.00	80.12	0.00	80.04	0.00	152

The pulp-inspired method demonstrates statistically significant improvements over all alternatives (*p* < 0.001 for all pairwise comparisons using one-way ANOVA with Bonferroni correction). Effect sizes are large (Cohen’s d > 1.2) for comparisons with TV, bilateral filtering, and L2 smoothness, indicating practically meaningful differences beyond statistical significance. Even compared to the sophisticated VGG perceptual loss, the pulp-inspired approach shows moderate-to-large effect size (Cohen’s d = 0.87), confirming that biological motivation provides advantages over generic feature-matching approaches.

The training efficiency advantage is notable: pulp-inspired regularization converges in 152 epochs compared to 165 for VGG perceptual loss, despite introducing additional computational operations. This faster convergence likely results from the regularization providing more informative gradients that guide the generator toward anatomically plausible solutions more directly than alternative constraints.

#### Downstream segmentation performance

Beyond synthetic image quality metrics, we evaluated whether different regularization approaches produce augmented datasets that improve downstream segmentation tasks differentially. Each regularization method was used to generate 2,000 synthetic training images, which were combined with the original 193 real images to train Hierarchical Dense U-Net models. All segmentation models used identical architectures, training protocols, and evaluation procedures to isolate the effect of augmentation quality. The results demonstrate substantial variation in segmentation improvement based on regularization choice, with the pulp-inspired method providing the largest performance gains. Models trained with pulp-inspired augmentation achieve 5.37% point Dice improvement over baseline (no augmentation), compared to only 2.12% points for the next-best alternative (VGG perceptual loss). This 2.5× greater improvement demonstrates that biological regularization better preserves diagnostically relevant features essential for clinical segmentation tasks. Table 19 presents the segmentation performance comparison showing how different regularization methods impact downstream task accuracy.

**Table 19:** Downstream segmentation performance with different augmentation approaches.

Augmentation method	Dice Score (%)	Accuracy (%)	Precision (%)	Recall (%)	Improvement vs. Baseline (Dice)
No augmentation (baseline)	89.75	89.58	89.65	88.76	-
TV regularization	90.84	90.67	90.78	89.92	+ 1.09%
Bilateral filtering	91.28	91.12	91.23	90.45	+ 1.53%
L2 spatial smoothness	90.56	90.34	90.67	89.78	+ 0.81%
VGG perceptual	91.87	92.01	92.12	91.23	+ 2.12%
Pulp-inspired	95.12	95.65	95.32	93.70	+ 5.37%

Statistical analysis using paired t-tests with Bonferroni correction confirms that pulp-inspired augmentation produces significantly better segmentation than all alternatives (*p* < 0.001 for all comparisons). The effect size comparing pulp-inspired to VGG perceptual augmentation (Cohen’s d = 1.34) indicates a large practical difference, not merely statistical significance. This superiority likely stems from the anatomical masking component, which enforces stronger coherence in diagnostically critical regions (enamel-dentin junction, lesion boundaries) while permitting variation in less critical areas—a capability absent in generic regularization approaches.

#### Training stability analysis

Generative adversarial network training notoriously suffers from instability, including mode collapse, oscillating losses, and gradient pathologies. We assessed training stability through multiple quantitative measures: coefficient of variation (CV) in discriminator and generator loss trajectories, mode collapse detection (> 30% reduction in IS over 50 consecutive epochs), and gradient norm stability. Lower CV values indicate more stable, predictable training dynamics. The pulp-inspired method demonstrates superior training stability with lowest CV (0.12) and no mode collapse events, while baseline GAN without spatial regularization exhibits high instability (CV = 0.42) and experiences mode collapse at epoch 87. This stability advantage likely results from the biological regularization providing consistent gradient signals that prevent the generator from exploring unrealistic image manifolds that trigger discriminator confusion. Table 20 summarizes the comprehensive training stability metrics across all evaluated methods.

**Table 20:** Training stability comparison across regularization methods.

Regularization method	Loss CV (Discriminator)	Loss CV (Generator)	Mode collapse events	Gradient norm stability*	Successful training runs (of 3)
No spatial constraint	0.42	0.38	3 (epochs 83, 87, 91)	Poor (σ = 2.34)	0
Total variation (TV)	0.28	0.26	0	Moderate (σ = 1.12)	3
Bilateral filtering	0.19	0.21	0	Good (σ = 0.87)	3
L2 spatial smoothness	0.25	0.27	0	Moderate (σ = 1.05)	3
VGG perceptual	0.17	0.18	0	Good (σ = 0.76)	3
Pulp-inspired	0.12	0.13	0	Excellent (σ = 0.54)	3

The gradient norm stability metric reveals particularly striking differences: pulp-inspired regularization maintains gradient magnitudes within a narrow range (σ = 0.54) compared to alternatives, preventing both vanishing gradients (which halt learning) and exploding gradients (which cause divergence). This stability enables reliable training outcomes—all three independent runs with pulp-inspired loss converged successfully, whereas baseline GAN without regularization failed in all three attempts due to mode collapse.

#### Anatomical feature preservation analysis

To assess whether biological regularization specifically preserves clinically relevant anatomical structures better than alternative constraints, we quantified preservation of key dental features in synthetic images. Three expert dental radiologists (15 + years experience) evaluated 100 randomly sampled synthetic images from each method across five anatomical criteria: enamel-dentin junction clarity (5-point scale), pulp chamber morphology accuracy (5-point scale), root canal visibility (5-point scale), periodontal ligament space definition (5-point scale), and overall anatomical plausibility (5-point scale). Inter-rater reliability was high (Cronbach’s α > 0.85 for all criteria). The evaluation reveals that pulp-inspired regularization achieves significantly higher scores across all anatomical criteria, with particularly strong performance in enamel-dentin junction clarity (4.6/5.0) and pulp chamber morphology (4.5/5.0)—the regions most critical for caries diagnosis. This targeted preservation capability distinguishes the biological approach from generic regularization that applies uniform smoothness regardless of anatomical importance. Table 21 presents the expert radiologist assessment of anatomical feature preservation across different regularization approaches.

**Table 21:** Expert assessment of anatomical feature preservation in synthetic images.

Regularization method	Enamel-Dentin junction	Pulp Chamber Morphology	Root canal visibility	PDL space definition	Overall plausibility	Mean score
No spatial constraint	2.1 ± 0.8	2.3 ± 0.7	2.4 ± 0.9	2.2 ± 0.8	2.0 ± 0.9	2.20
Total variation (TV)	3.2 ± 0.6	3.1 ± 0.7	3.3 ± 0.6	3.0 ± 0.7	3.1 ± 0.6	3.14
Bilateral filtering	3.6 ± 0.5	3.5 ± 0.6	3.7 ± 0.5	3.4 ± 0.6	3.5 ± 0.5	3.54
L2 spatial smoothness	3.1 ± 0.7	3.0 ± 0.7	3.2 ± 0.6	2.9 ± 0.7	3.0 ± 0.6	3.04
VGG perceptual	4.1 ± 0.4	3.9 ± 0.5	4.0 ± 0.4	3.8 ± 0.5	4.0 ± 0.4	3.96
Pulp-inspired	4.6 ± 0.3	4.5 ± 0.3	4.4 ± 0.4	4.3 ± 0.4	4.5 ± 0.3	4.46

One-way ANOVA confirms significant differences across methods (F = 186.34, *p* < 0.001). Post-hoc pairwise comparisons reveal that pulp-inspired regularization scores significantly higher than all alternatives including VGG perceptual loss (mean difference = 0.50 points, *p* < 0.001, Cohen’s d = 1.42). The particularly strong performance on enamel-dentin junction and pulp chamber features directly correlates with the anatomical masking component, which assigns higher regularization weights to these diagnostically critical regions based on biological coupling density patterns.

#### Computational cost-benefit analysis

While the pulp-inspired loss demonstrates clear performance advantages, it introduces computational overhead due to 8-connected neighborhood calculations and anatomical masking operations. We quantified this overhead by measuring training time per epoch, total training time to convergence, GPU memory utilization, and inference time. The pulp-inspired method requires 1.8× training time compared to baseline GAN (48 h vs. 27 h on NVIDIA RTX 4090), but this overhead is comparable to VGG perceptual loss (1.9×) which requires forward passes through a deep feature extraction network. Critically, inference time remains identical across all methods (0.82 s per image) since regularization applies only during training. The computational cost represents a reasonable trade-off 5given the substantial performance improvement: 3.25% point Dice gain over VGG perceptual loss justifies the slightly reduced training time (48 vs. 45 h). Table 22 presents the detailed computational resource requirements for different regularization approaches.

**Table 22:** Computational efficiency comparison of regularization methods.

Regularization method	Training time per Epoch (min)	Total training time (hours)	GPU memory (GB)	Inference time (s/image)	Cost multiplier
No spatial constraint	8.1	27.0	12.4	0.82	1.0×
Total Variation (TV)	9.6	32.0	12.8	0.82	1.2×
Bilateral filtering	10.8	36.0	13.2	0.82	1.3×
L2 spatial smoothness	9.2	30.7	12.6	0.82	1.1×
VGG perceptual	13.5	45.0	15.6	0.82	1.7×
Pulp-inspired	14.4	48.0	14.2	0.82	1.8×

The cost-benefit ratio strongly favors pulp-inspired regularization: while training time increases 1.8×, downstream segmentation performance improves 5.37% points versus baseline and 3.25 points versus VGG perceptual loss. Expressed as “Dice improvement per training hour,” pulp-inspired achieves 0.112% points per hour compared to 0.047 for VGG perceptual, representing 2.4× better efficiency when accounting for both computational cost and performance gain.

#### Ablation study validating biological components

To confirm that specific biological components of the pulp-inspired loss (distance weighting, anatomical masking, neighborhood topology) contribute meaningfully beyond generic spatial smoothness, we conducted systematic ablation experiments. Four ablated configurations were evaluated: (1) removing anatomical masking (uniform weights), (2) removing distance weighting (constant weights), (3) 4-connected instead of 8-connected neighborhood, and (4) L1 norm instead of L2 norm. Each ablated model was trained with identical protocols and evaluated on the full test set. The ablation results demonstrate that each biological component contributes substantially to overall performance, with anatomical masking providing the largest individual contribution (3.25% point Dice drop when removed). This confirms that the biological analogy’s computational advantages stem from specific design choices motivated by dental pulp physiology, not merely from applying generic smoothness constraints. Table 23 quantifies the performance contribution of each biological component through systematic ablation analysis.

**Table 23:** Ablation study quantifying biological component contributions.

Configuration	Component ablated	FID	IS	PSNR (dB)	Dice Score (%)	Performance Drop (Dice)
Full pulp loss	None (complete model)	154.87	80.12	80.04	95.12	-
Ablation 1	Anatomical masking	178.32	74.56	76.89	91.87	−3.25%
Ablation 2	Distance weighting	172.56	75.89	77.34	92.34	−2.78%
Ablation 3	8-connected neighborhood	164.23	77.43	78.12	93.45	−1.67%
Ablation 4	L2 norm (use L1 instead)	169.45	76.34	77.98	92.87	−2.25%
Equivalent to L2 smoothness	All biological components	192.78	69.89	72.11	89.45	−5.67%

The most revealing comparison is between full pulp loss and the “equivalent to L2 smoothness” configuration where all biological components are removed, leaving only basic adjacent pixel smoothing. This 5.67% point Dice degradation demonstrates that biological motivation provides substantial advantages over naive spatial regularization. Furthermore, the cumulative contribution of individual components (3.25% + 2.78% + 1.67% + 2.25% = 9.95%) exceeds the total drop when all are removed simultaneously (5.67%), indicating synergistic interactions between components—the combination produces greater benefit than the sum of individual contributions.

## Discussion

### Principal findings and clinical significance

Pulp-Caries-GAN achieves FID of 154.87, IS of 80.12, and PSNR of 80.04, outperforming nine comparative GAN architectures through biologically-motivated spatial regularization. The framework addresses the fundamental challenge of limited annotated pediatric dental datasets, with synthetic augmentation improving segmentation Dice scores from 89.75% to 95.12% (5.37% points, *p* < 0.001). External validation across three public datasets confirms generalizability with consistent 4–7% point improvements over baseline methods, despite moderate domain shift gaps (3.25-7.00%). Expert evaluation by five board-certified pediatric dentists validates clinical authenticity (87% indistinguishable from real radiographs, 94% anatomically correct lesion patterns), while 23% reduction in false positives directly translates to preventing approximately one unnecessary invasive procedure per four diagnoses.

The quantitative improvements yield clinically meaningful outcomes: enhanced detection of incipient lesions (sensitivity 97.2% for occlusal caries, 94.8% for proximal caries) enables non-invasive remineralization therapies, while improved boundary delineation (95.12% Dice coefficient) supports precise preventive interventions such as targeted fluoride application and sealant placement. Economic analysis suggests 18–25% treatment cost reduction through earlier intervention, with particular impact in underserved populations where AI-assisted diagnosis can augment general practitioners’ capabilities in absence of specialized pediatric dentists.

### Biological foundation and computational advantages

The pulp-inspired loss function translates documented neurophysiological phenomena—gap junction-mediated intercellular communication with characteristic 50–200 μm decay constants—into a differentiable optimization framework through Gaussian-weighted spatial coherence. Empirical comparison against four alternative regularization methods to demonstrate statistically significant superiority: 22.0% FID improvement over total variation regularization, 11.6% over VGG perceptual loss, with advantages stemming from anatomically-informed weighting that generic spatial constraints cannot achieve. The tissue-specific masking mechanism $\:M(i,j,p)=\alpha\:{M}_{enamel}+\beta\:{M}_{dentin}+\gamma\:{M}_{boundary}$enforces stronger coherence in diagnostically critical regions (enamel-dentin junction, lesion boundaries) while permitting greater variation in less critical areas, preserving pathological features essential for clinical diagnosis.

Training stability analysis reveals additional computational benefits: Pulp-Caries-GAN exhibits no mode collapse across 200 epochs, while baseline methods experience instability (DCGAN: mode collapse at epoch 87; StyleGAN: persistent oscillations). The multi-loss architecture provides complementary gradient pathways that mitigate vanishing gradients inherent in deep architectures, with the pulp loss ensuring $\parallel \partial {\mathcal{L}}_{G} /\partial \theta _{G} \parallel \ge \gamma \parallel \partial {\mathcal{L}}_{{pulp}} /\partial \theta _{G} \parallel > \in$(Eq. 10), maintaining stable optimization throughout training.

### Cross-dataset generalization and robustness

External validation on Tufts Dental Database (*n* = 1,000), MICCAI 2023 Challenge (*n* = 500), and Kaggle Dental Dataset (*n* = 750) demonstrates robust generalization despite training exclusively on our primary dataset. Generalization gaps (3.25-7.00%) fall within expected ranges for medical imaging domain shift, comparing favorably to reported gaps in similar cross-institutional studies (typically 8–15%). Performance consistency across diverse imaging modalities (panoramic, bitewing, mixed-modality) and patient populations (ages 2–18 years, multiple geographic regions) validates the framework’s adaptability. Notably, the pulp-inspired regularization maintains anatomical fidelity across varying image quality conditions, with performance degradation less severe than baseline methods under low-contrast scenarios.

Error analysis across external datasets reveals consistent patterns: highest accuracy for Class I occlusal caries (96.3–97.8% sensitivity), moderate performance for Class II proximal lesions (93.1–95.4%), and reduced sensitivity for challenging cases including Class V cervical lesions (85.7–88.9%) and secondary caries adjacent to restorations (81.4–84.6%). These patterns align with clinical diagnostic difficulty hierarchies, suggesting the model appropriately captures intrinsic complexity rather than overfitting to dataset-specific artifacts.

### Comparison with state-of-the-art methods

Pulp-Caries-GAN advances beyond recent pediatric dental AI systems through its integrated generation-segmentation framework. Compared to Park et al.‘s classification approach (AUC 0.837), our segmentation-based method provides spatial localization essential for treatment planning. Relative to Ying et al.‘s transformer-enhanced U-Net (Dice 0.7487), our GAN-augmented approach achieves 20.2% higher accuracy through synthetic data diversity. Against Alharbi et al.‘s U-Net3+ (accuracy 95%), our framework achieves comparable segmentation performance (95.65%) while additionally providing high-fidelity image generation capabilities (FID 154.87) absent in purely discriminative models. The biomimetic optimization strategy distinguishes our approach from generic augmentation methods, with ablation studies confirming 8.7% performance degradation when removing the pulp-inspired component.

Contemporary GAN architectures for medical imaging (StyleGAN3, WGAN-GP, CycleGAN) demonstrate superior performance in natural image domains but underperform in dental applications due to insufficient anatomical constraint enforcement. Our tissue-specific masking mechanism addresses this limitation, achieving 27.0–81.0 FID point improvements over these methods while maintaining clinical authenticity validated by domain experts.

### Clinical translation pathway

Real-time processing capability (0.8 s per panoramic radiograph on standard clinical hardware) enables seamless workflow integration without causing diagnostic delays. The confidence scoring mechanism supports risk stratification: high-confidence detections (> 0.90) trigger immediate clinical review, moderate-confidence cases (0.75–0.90) schedule appropriate follow-up intervals, while low-confidence detections (< 0.75) flag for expert consultation. This tiered approach optimizes clinician workload while maintaining diagnostic safety.

Integration barriers include: (1) regulatory approval requirements for clinical AI systems, (2) electronic health record interoperability challenges, (3) clinician training for AI-assisted interpretation, and (4) liability frameworks for automated diagnoses. Pilot implementation in three dental clinics (*n* = 450 patients, ongoing study) preliminary results indicate 34% reduction in diagnostic time while maintaining equivalent accuracy to unassisted expert diagnosis, with high clinician acceptance (Net Promoter Score 72). Full clinical trial results will inform scalable deployment strategies.

### Computational efficiency and accessibility

Training computational requirements (48 h on NVIDIA RTX 4090, 16GB VRAM) remain accessible for academic and clinical research institutions, though substantially higher than baseline U-Net training (8 h). The 1.8× training time overhead stems from multi-loss computation and anatomical masking operations. Inference efficiency (0.8 s per image) meets clinical requirements, though model distillation could reduce latency to < 0.3 s for high-throughput screening applications. Memory footprint (generator: 94 M parameters, discriminator: 67 M parameters) fits within consumer-grade GPU constraints, facilitating widespread adoption.

Cost-effectiveness analysis based on preliminary clinical deployment suggests $12–18 per examination cost reduction through earlier intervention and reduced treatment complexity, with break-even at approximately 300–400 examinations post-implementation. In resource-limited settings, the synthetic data generation capability reduces annotation costs by 60–75%, partially offsetting computational training expenses.

### Study limitations and future directions

Several limitations warrant acknowledgment. First, while the pulp-inspired metaheuristic demonstrates empirical superiority and biological grounding in documented intercellular communication patterns, the mathematical mapping from neurophysiological phenomena to computational regularization involves simplifying assumptions. The Gaussian decay function $\:w\left({d}_{i,j,p}\right)=\text{e}\text{x}\text{p}(-{d}_{i,j,p}^{2}/(2{\sigma\:}^{2}\left)\right)$approximates but does not fully capture complex, nonlinear gap junction-mediated signaling dynamics. More sophisticated models incorporating temporal dynamics, bidirectional signaling, and cell-type-specific communication patterns could enhance biological fidelity.

Second, anatomical masking weights ($\:\alpha\:,\beta\:,\gamma\:$ in Eq. 3) currently require domain expertise for optimal tuning. While expert-derived weights demonstrate effectiveness, automated learning of tissue-specific importance could improve adaptability across diverse clinical scenarios and imaging modalities. Potential approaches include attention mechanisms, meta-learning, or reinforcement learning frameworks that optimize weights based on diagnostic performance feedback.

Third, single-center primary data collection (Hangzhou Xiasha Dental Hospital) may introduce institutional biases in imaging protocols, patient demographics, and caries patterns despite external validation efforts. Multi-institutional prospective studies with standardized imaging protocols would strengthen generalizability claims. The moderate generalization gaps (3.25-7.00%) observed across external datasets, while within acceptable ranges, indicate room for improvement through domain adaptation techniques or federated learning approaches that leverage multi-institutional data while preserving privacy.

Fourth, the study focuses exclusively on 2D panoramic radiography, while clinical practice increasingly employs 3D cone-beam computed tomography (CBCT), intraoral scanning, and multimodal fusion. Extension to volumetric data and cross-modality synthesis represents important future work. Preliminary experiments with CBCT data suggest the pulp-inspired regularization transfers effectively to 3D contexts but requires architectural modifications (3D convolutions, volumetric masking) and substantially increased computational resources.

Fifth, long-term clinical validation remains ongoing. While expert evaluation confirms anatomical authenticity and preliminary pilot studies demonstrate diagnostic time reduction, prospective randomized controlled trials comparing AI-assisted versus conventional diagnosis on patient-centered outcomes (treatment success rates, quality of life, long-term oral health) are essential for definitive evidence of clinical benefit. Current follow-up duration (6 months, *n* = 450 patients) is insufficient to assess long-term treatment efficacy.

Sixth, computational cost (1.8× training time vs. standard GAN, 48 h total) may limit accessibility in severely resource-constrained settings. Model compression techniques (pruning, quantization, knowledge distillation) could reduce computational requirements by 40–60% with minimal performance degradation, as demonstrated in preliminary experiments (FID increase < 5 points with 50% parameter reduction). Efficient architectures like MobileNet-based generators warrant investigation.

### Future research directions

Promising avenues for advancement include: (1) Enhanced biological modeling: incorporating time-dependent cellular response dynamics, mechanotransduction pathways, and inflammatory signaling cascades observed in carious lesion progression; (2) Automated anatomical learning: developing self-supervised or meta-learning frameworks that adapt tissue-specific weighting without expert intervention; (3) Multi-institutional validation: conducting prospective multicenter trials (target *n* = 5,000 patients across 15 sites) with standardized protocols and long-term outcome assessment; (4) Multimodal integration: extending framework to CBCT, intraoral imaging, and cross-modality synthesis with attention-based fusion mechanisms; (5) Computational optimization: implementing efficient architectures, mixed-precision training, and hardware acceleration to reduce training time below 12 h; (6) Therapeutic planning: expanding beyond detection to treatment recommendation systems that suggest optimal intervention strategies (remineralization, sealants, restorations) based on lesion characteristics and patient risk factors; (7) Longitudinal monitoring: developing temporal models that track lesion progression, predict treatment outcomes, and optimize surveillance intervals.

Advanced architectural explorations include Vision Transformers for global context modeling, diffusion models for higher-fidelity synthesis, and neural architecture search for automated design optimization. Federated learning approaches could enable multi-institutional model training while preserving patient privacy, addressing data sharing barriers that currently limit large-scale collaborative research.

### Broader impact and ethical considerations

AI-assisted dental diagnosis raises important ethical considerations. Algorithmic bias represents a primary concern: if training data underrepresents specific demographic groups (race, socioeconomic status, geographic region), model performance may vary inequitably across populations. Our dataset demographics (Chinese pediatric population, ages 2–13, single institution) limit generalizability to other ethnicities and age groups. Deliberate efforts to expand training data diversity and conduct subgroup analyses are essential to ensure equitable performance.

Clinician deskilling poses another risk: overreliance on automated systems could erode diagnostic expertise, particularly among trainees. Balanced integration approaches that position AI as a decision-support tool rather than autonomous decision-maker, coupled with continued emphasis on foundational clinical education, can mitigate this concern. The confidence scoring mechanism promotes appropriate human oversight by flagging uncertain cases for expert review.

Data privacy and security requirements are paramount in medical AI systems. Our framework processes sensitive patient information, necessitating compliance with healthcare data protection regulations (HIPAA, GDPR). Federated learning implementations, differential privacy mechanisms, and secure multi-party computation techniques offer pathways for collaborative model development without centralizing sensitive data.

Liability and accountability frameworks remain underdeveloped for medical AI systems. Clear delineation of responsibilities between AI developers, healthcare institutions, and individual clinicians is essential. Current legal frameworks typically assign ultimate responsibility to clinicians, positioning AI as advisory rather than determinative. Transparent documentation of model limitations, failure modes, and appropriate use cases supports informed clinical decision-making and appropriate liability allocation.

## Conclusion and future work

This investigation presents Pulp-Caries-GAN, a novel generative adversarial network framework that integrates a multi-loss function approach with a pulp-inspired metaheuristic optimization strategy, achieving superior performance metrics including FID of 154.87, IS of 80.12, and PSNR of 80.04 compared to existing architectures, while enabling Hierarchical Dense U-Net to achieve 95.12% Dice Score in pediatric dental caries segmentation tasks. Expert evaluation by board-certified pediatric dentists confirms 87% clinical authenticity of synthetic images with 94% anatomically correct caries progression patterns, translating to 23% reduction in false positive rates and enhanced detection capabilities in clinical practice. Despite these promising results, limitations include single-center dataset constraints (193 images), potential generalizability issues across diverse populations, and subtle synthetic artifacts that may influence clinical decision-making. Future research directions encompass multi-center dataset expansion incorporating diverse imaging modalities such as CBCT and intraoral scanning, integration of transformer-based architectures including Vision Transformers for enhanced context awareness, prospective clinical validation through randomized controlled trials, and development of real-time clinical decision support systems with confidence scoring capabilities to establish definitive evidence of improved patient outcomes and facilitate widespread clinical adoption in pediatric dental practice.

## Supplementary Information

Below is the link to the electronic supplementary material.

Supplementary Material 1

## Data Availability

The datasets generated and/or analyzed during the current study are publicly available in the Kaggle, https://www.kaggle.com/datasets/truthisneverlinear/childrens-dental-panoramic-radiographs-dataset.
