# Supplementary material for: A novel Pulp Caries GAN multi loss GAN with new pulp inspired metaheuristics for pediatric dental caries detection and segmentation
Source: Sci Rep. 2026 Jan 8;16:875. doi: 10.1038/s41598-025-28459-8 (PMC12783627; doi:10.1038/s41598-025-28459-8)
Supplement: Supplementary file 1 — Supplementary Material 1 [file 41598_2025_28459_MOESM1_ESM.docx]

Table S1 presents the descriptive statistics for the Fréchet Inception Distance (FID) scores of various GAN architectures evaluated on the pediatric dental caries dataset. The mean FID score across these models is 214.77, with a standard deviation of 44.66, indicating a diverse performance range among the architectures. The variance of 1994.91 reflects the spread of the scores, with a minimum score of 154.87 achieved by Pulp-caries-GAN and a maximum score of 284.98 by the traditional GAN model. The 95% confidence interval for the mean FID score ranges from 190.04 to 239.51, suggesting that most models fall within this range. Importantly, the Pulp-caries-GAN, with its lower score of 154.87, not only stands out as the most effective model but also emphasizes the effectiveness of our methodology in generating high-quality images for pediatric dental diagnostics. These statistics underscore the robustness of Pulp-caries-GAN in achieving superior image generation compared to its counterparts, further supporting its application in clinical settings.

**Table S1:** Descriptive statistics of FID-based comparison of GAN architectures across pediatric dental caries dataset

| **Statistic** | **Pediatric Dental Caries Dataset** |
| --- | --- |
| Mean | 214.77 |
| Std. Deviation | 44.66 |
| Variance | 1994.91 |
| Minimum | 154.87 |
| Maximum | 284.98 |
| 95% Confidence Interval of Mean | 190.04 - 239.51 |
| Mean ± Std. | 214.77 ± 44.66 |

Table S2 provides the descriptive statistics for the Inception Score (IS) of various GAN architectures evaluated on the pediatric dental caries dataset. The mean IS across these models is 71.76, with a standard deviation of 5.87, indicating a relatively consistent performance among the architectures. The variance of 34.51 reflects the degree of variability in their scores, with Pulp-caries-GAN achieving the maximum score of 80.12, while the lowest score recorded is 60.87. The 95% confidence interval for the mean IS ranges from 68.51 to 75.02, suggesting that most models cluster within this range. Importantly, the superior performance of Pulp-caries-GAN, with its exceptional score of 80.12, highlights the effectiveness of our methodology in generating diverse and high-quality images suitable for pediatric dental applications. This analysis reveals that Pulp-caries-GAN not only meets but exceeds the performance of its peers, reinforcing its potential as a valuable tool in clinical practice.

**Table S2:** Descriptive statistics of IS-based comparison of GAN architectures across pediatric dental caries dataset

| **Statistic** | **Pediatric Dental Caries Dataset** |
| --- | --- |
| Mean | 71.76 |
| Std. Deviation | 5.87 |
| Variance | 34.51 |
| Minimum | 60.87 |
| Maximum | 80.12 |
| 95% Confidence Interval of Mean | 68.51 - 75.02 |
| Mean ± Std. | 71.76 ± 5.87 |

Table S3 provides the descriptive statistics for the PSNR values of various GAN architectures evaluated on the pediatric dental caries dataset. The mean PSNR across these models is 72.05, with a standard deviation of 4.82, indicating a consistent level of performance among the architectures. The variance of 23.26 reflects the variability in PSNR scores, with a minimum score of 64.86 and a maximum score of 80.04 achieved by Pulp-caries-GAN. The 95% confidence interval for the mean PSNR ranges from 69.38 to 74.72, suggesting that most models fall within this range. Notably, the exceptional performance of Pulp-caries-GAN, with its score of 80.04, highlights the effectiveness of our methodology in generating high-quality images suitable for pediatric dental diagnostics. These statistics reinforce the significance of Pulp-caries-GAN as a leading model in this domain, emphasizing its potential to enhance diagnostic accuracy and treatment outcomes in pediatric dentistry.

Table S3: Descriptive statistics of PSNR-based comparison of GAN architectures across pediatric dental caries dataset

| **Statistic** | **Pediatric Dental Caries Dataset** |
| --- | --- |
| Mean | 72.05 |
| Std. Deviation | 4.82 |
| Variance | 23.26 |
| Minimum | 64.86 |
| Maximum | 80.04 |
| 95% Confidence Interval of Mean | 69.38 - 74.72 |
| Mean ± Std. | 72.05 ± 4.82 |

Table S4 presents the descriptive statistics for the segmentation results of pediatric dental caries before the application of Pulp-caries-GAN. The mean Dice Score is 87.75, with a standard deviation of 1.26, indicating a high level of consistency across the models evaluated. The mean Accuracy is 87.67, while the Precision and Recall are 87.54 and 87.60, respectively. The variance in these metrics is relatively low, with values of 1.59 for Dice Score, 1.61 for Accuracy, 2.17 for Precision, and 1.36 for Recall, suggesting that the models demonstrate a consistent performance in segmenting dental caries. The minimum values recorded are 85.76 for the Dice Score and 85.62 for Accuracy, while the maximum values reached are 89.75 for the Dice Score and 89.58 for Accuracy, reflecting the effectiveness of the top-performing models. The 95% confidence intervals for the mean metrics indicate that the Dice Score ranges from 86.85 to 88.66, the Accuracy from 86.76 to 88.58, the Precision from 86.49 to 88.60, and the Recall from 86.76 to 88.44. This analysis underscores the strong performance of the segmentation models, laying a solid foundation for the subsequent image generation process by Pulp-caries-GAN.

Table S4: Descriptive statistics of segmentation results of pediatric dental caries before applying Pulp-caries GAN

| **Statistic** | **Dice Score (%)** | **Accuracy (%)** | **Precision (%)** | **Recall (%)** |
| --- | --- | --- | --- | --- |
| Mean | 87.75 | 87.67 | 87.54 | 87.60 |
| Std. Deviation | 1.26 | 1.27 | 1.47 | 1.17 |
| Variance | 1.59 | 1.61 | 2.17 | 1.36 |
| Minimum | 85.76 | 85.62 | 85.09 | 85.86 |
| Maximum | 89.75 | 89.58 | 89.65 | 89.41 |
| 95% Confidence Interval of Mean | 86.85 - 88.66 | 86.76 - 88.58 | 86.49 - 88.60 | 86.76 - 88.44 |
| Mean ± Std. | 87.75 ± 1.26 | 87.67 ± 1.27 | 87.54 ± 1.47 | 87.60 ± 1.17 |

Table S5 presents the results of the Bonferroni Post-hoc Tests for the segmentation metrics of pediatric dental caries before applying Pulp-caries-GAN. The mean differences among the various metrics, including Dice Score, Accuracy, Precision, and Recall, show minimal variation. For example, the mean difference between Dice Score and Accuracy is 0.08, with a standard error of 0.58 and a corresponding p-value of 1, indicating no statistically significant difference. Similarly, the comparisons between Dice Score and Precision (mean difference of 0.21), Dice Score and Recall (mean difference of 0.15), and Accuracy and Precision (mean difference of 0.13) all yielded p-values of 1, reflecting a lack of significant differences among these metrics. The confidence intervals for these comparisons further reinforce the results, with lower and upper limits indicating no substantial differences. In the comparison between Precision and Recall, a mean difference of -0.06 was observed, again with a p-value of 1. These results suggest that the segmentation metrics are closely aligned in performance, underscoring the effectiveness of the models used in this study. Overall, the Bonferroni Post-hoc Tests indicate that while the models perform well, there are no significant differences in their segmentation capabilities.

**Table S5:** Bonferroni Post-hoc Tests for segmentation results of pediatric dental caries before applying Pulp-caries GAN

| **Comparison (I)** | **Comparison (J)** | **Mean Difference** | **Std. Error** | **t** | **p** | **95% CI Lower Limit** | **95% CI Upper Limit** |
| --- | --- | --- | --- | --- | --- | --- | --- |
| Dice Score | Accuracy | 0.08 | 0.58 | 0.14 | 1 | -1.57 | 1.74 |
| Dice Score | Precision | 0.21 | 0.58 | 0.36 | 1 | -1.45 | 1.86 |
| Dice Score | Recall | 0.15 | 0.58 | 0.26 | 1 | -1.50 | 1.81 |
| Accuracy | Precision | 0.13 | 0.58 | 0.22 | 1 | -1.53 | 1.78 |
| Accuracy | Recall | 0.07 | 0.58 | 0.12 | 1 | -1.59 | 1.72 |
| Precision | Recall | -0.06 | 0.58 | -0.10 | 1 | -1.71 | 1.60 |

Table S6 presents the descriptive statistics for the segmentation results of pediatric dental caries after the application of Pulp-caries-GAN. The mean Dice Score is 92.88, reflecting a substantial improvement in segmentation accuracy compared to previous evaluations. The mean Accuracy is 93.08, with a standard deviation of 1.92, indicating a high level of consistency among the models.

The metrics also reveal a mean Precision of 92.99 and a Recall of 92.86, both demonstrating strong performance. The variance is relatively low, with values of 3.03 for Dice Score, 3.68 for Accuracy, 3.99 for Precision, and 3.42 for Recall, suggesting that the models exhibit consistent performance across metrics.

The minimum values recorded are 90.65 for the Dice Score and 90.64 for Accuracy, while the maximum values achieved are 95.12 for the Dice Score and 95.65 for Accuracy. The 95% confidence intervals for the mean metrics indicate that the Dice Score ranges from 91.64 to 94.13, Accuracy from 91.70 to 94.45, Precision from 91.56 to 94.41, and Recall from 91.54 to 94.18. These statistics highlight the significant enhancement in segmentation capabilities following the application of Pulp-caries-GAN, reinforcing its value in pediatric dental diagnostics and treatment planning.

Table S6: Descriptive statistics of segmentation results of pediatric dental caries after applying Pulp-caries GAN

| **Statistic** | **Dice Score (%)** | **Accuracy (%)** | **Precision (%)** | **Recall (%)** |
| --- | --- | --- | --- | --- |
| Mean | 92.88 | 93.08 | 92.99 | 92.86 |
| Std. Deviation | 1.74 | 1.92 | 2.00 | 1.85 |
| Variance | 3.03 | 3.68 | 3.99 | 3.42 |
| Minimum | 90.65 | 90.64 | 90.29 | 90.45 |
| Maximum | 95.12 | 95.65 | 95.54 | 95.43 |
| 95% Confidence Interval of Mean | 91.64 - 94.13 | 91.70 - 94.45 | 91.56 - 94.41 | 91.54 - 94.18 |
| Mean ± Std. | 92.88 ± 1.74 | 93.08 ± 1.92 | 92.99 ± 2.00 | 92.86 ± 1.85 |

Table S7 presents the results of the Bonferroni Post-hoc Tests for the segmentation metrics of pediatric dental caries after applying Pulp-caries-GAN. The mean differences among the various metrics—Dice Score, Accuracy, Precision, and Recall—indicate minimal variation. For example, the mean difference between Dice Score and Accuracy is -0.19, with a standard error of 0.84 and a corresponding ppp-value of 1, suggesting no statistically significant difference between these two metrics.

Similar results are observed in the comparisons between Dice Score and Precision (mean difference of -0.10) and Dice Score and Recall (mean difference of 0.02), both yielding ppp-values of 1. Other comparisons, such as Accuracy versus Precision (mean difference of 0.09) and Accuracy versus Recall (mean difference of 0.22), also produced ppp-values of 1, indicating no significant differences among these metrics.

The confidence intervals for these comparisons further support the results, with lower and upper limits indicating a lack of substantial differences. Overall, the Bonferroni Post-hoc Tests show that while the segmentation metrics demonstrate strong performance after applying Pulp-caries-GAN, there are no significant differences in their capabilities, highlighting the effectiveness of the models used in this study.

**Table S7:** Bonferroni post-hoc tests for segmentation results of pediatric dental caries after applying Pulp-caries GAN

| **Comparison (I)** | **Comparison (J)** | **Mean Difference** | **Std. Error** | **t** | **p** | **95% CI Lower Limit** | **95% CI Upper Limit** |
| --- | --- | --- | --- | --- | --- | --- | --- |
| Dice Score | Accuracy | -0.19 | 0.84 | -0.23 | 1 | -2.59 | 2.20 |
| Dice Score | Precision | -0.10 | 0.84 | -0.12 | 1 | -2.50 | 2.29 |
| Dice Score | Recall | 0.02 | 0.84 | 0.03 | 1 | -2.37 | 2.42 |
| Accuracy | Precision | 0.09 | 0.84 | 0.11 | 1 | -2.30 | 2.48 |
| Accuracy | Recall | 0.22 | 0.84 | 0.26 | 1 | -2.18 | 2.61 |
| Precision | Recall | 0.13 | 0.84 | 0.15 | 1 | -2.27 | 2.52 |
